# Life strategies in an upwelling world: distribution patterns and niche partitioning of Calanidae copepods in the Benguela Current

**DOI:** 10.1038/s41598-026-39910-9

**Published:** 2026-02-22

**Authors:** Maya Bode-Dalby, Hanna Rittinghaus, Tarron Lamont, Hans M. Verheye, Sabrina Dorschner, Anna Schukat, Wilhelm Hagen, Holger Auel

**Affiliations:** 1 BreMarE - Bremen Marine Ecology, Marine Zoology, Universität Bremen, P.O. Box 330440, 28334 Bremen, Germany; 2 Helmholtz Centre for Polar and Marine Research, Alfred Wegener Institute, Bremerhaven, Germany; 3 Department of Forestry, Fisheries, and the Environment, Oceans and Coasts Research Branch, Victoria & Alfred Waterfront, PO Box 52126, Cape Town, 8002 South Africa; 4 Bayworld Centre for Research and Education, 5 Riesling Road, Constantia, Cape Town, 7806 South Africa; 5 Department of Biological Sciences, University of Cape Town, Rondebosch, Private Bag X3, Cape Town, 7701 South Africa; 6 Department of Oceanography and Nansen-Tutu Centre for Marine Environmental Research, University of Cape Town, Private Bag X3, Rondebosch, Cape Town, South Africa

**Keywords:** Zooplankton, Functional traits, Diversity, Abundance, Stable isotopes, Fatty acids, Ecology, Ecology, Ocean sciences

## Abstract

Climate change is expected to alter coastal upwelling systems, making it essential to understand their current ecological structure. This study investigates the distribution and niche partitioning of Calanidae copepods in relation to upwelling intensities during austral summer in the northern (nBUS) and southern (sBUS) Benguela Current upwelling subsystems, which differ in upwelling seasonality, oxygen minimum zones and fisheries production. The six occurring Calanidae species were separated into three size categories each with potentially similar prey-size spectra. Similar-sized species differed in at least one of their horizontal, vertical or trophic niches. The dominant copepod *Calanoides natalis* was associated with cold, chlorophyll *a*-rich shelf waters and showed high levels of diatom fatty acid markers. *Calanus agulhensis* peaked offshore in the sBUS, with copepodids C5 also found deeper than 200 m and, unexpectedly, offshore in the nBUS, likely transported there by aged Agulhas retroflection rings drifting towards the nBUS and mixing with Subantarctic Mode Water, indicated by co-occurrences with *Neocalanus tonsus*. Copepodids C5 of *C. agulhensis* had elevated amounts of wax esters, suggesting special life-cycle adaptations of potential energy storage. *Nannocalanus minor* and *Mesocalanus tenuicornis* were associated with warm, offshore waters, with *N. minor* being more abundant in the nBUS and *M. tenuicornis* in the sBUS. They co-occurred to some extent but showed differences in their vertical and trophic niches. Fine-scale niche partitioning among closely related, similar-sized species likely supports persistence and diversity in highly dynamic upwelling systems. It raises questions about winners or losers in response to severe environmental changes related to future climate scenarios and how these changes may propagate to commercially important fish populations.

## Introduction

Copepods of the family Calanidae occupy key ecological positions worldwide as prey for commercially important fish and thereby sustain large-scale fisheries production from Polar Oceans to subtropical Eastern Boundary Upwelling Systems (EBUS). To identify climate-change-driven alterations and their implications, we need to understand the dynamics of ecosystems. The Benguela Current Upwelling System (BUS) off the west coast of southern Africa is one of the four major EBUS in the world that are important in terms of global primary production and fisheries yield. The BUS is divided by the perennial Lüderitz upwelling cell at ~ 26°S in a northern (nBUS) and a southern (sBUS) subsystem based on different environmental conditions^1^. In the BUS, sardine (*Sardinops sagax*) and anchovy (*Engraulis encrasicolus*) stocks collapsed in the 1960s and 1980s, respectively^2,3^. The sardine stock in the nBUS never recovered and Cape horse mackerel (*Trachurus capensis*) became the ecologically and economically most important pelagic fish species in the nBUS^4–6^. In addition, pelagic goby (*Sufflogobius bibarbatus*), which are well adapted to hypoxic conditions, have become an important dietary item for higher trophic levels such as Cape fur seals, African penguins, and other seabirds^4,7,8^. In contrast to the nBUS, sardine stocks have recovered in the sBUS since the late 1980s and anchovies since the late 1990s. However, the bulk of sardine and anchovy spawners has shifted towards the east of Cape Agulhas since 1999^9^ and 1996^10,11^, respectively. These contrasting fisheries yields and community structures, despite similar primary production, may reflect differences in trophic transfer efficiencies, channeling energy and organic matter through the food web^12^.

Copepods are a major component of mesozooplankton communities, which play a key role in the pelagic food webs of the BUS linking primary producers to higher trophic levels^13^. Upwelling in the nBUS is more diffuse but typically strongest during the austral winter and spring, whereas in the sBUS it is more strongly pulsed and peaks during the austral summer^1,13^. Seasonal changes in the latitudinal position of the high pressure and wind system cause these differences in the upwelling regimes. Similarly, copepod biomass in the nBUS shows a diffuse seasonal signal with high interannual variability, whereas in the sBUS it generally peaks in late summer^1,13,14^. For instance, around Walvis Bay (23°S) copepod abundance may peak in early summer months^15^, but at 20°S it is usually high in July, November/December and March to May with no obvious seasonal cycle^16^.

The upwelling water masses in the nBUS and sBUS are comprised of differing proportions of South Atlantic Central Water (SACW) and eastern South Atlantic Central Water (ESACW)^17,18^. SACW is nutrient- and CO_2_ rich but O_2_ depleted due to its long route through the South Atlantic Subtropical Gyre followed by the equatorial current system and the Angola Dome. In comparison, ESACW is younger, well oxygenated but contains less nutrients. Its source waters originate from the Indian Ocean (via the Agulhas retroflection), the Cape Basin, the Brazil-Malvinas Confluence, the South Atlantic Current, as well as from waters formed just north of the Subantarctic Front that subduct into the South Atlantic Subtropical Gyre. ESACW is the dominant upwelling water mass in the sBUS and during winter months in the nBUS. During summer, SACW spreads southward into the nBUS via a poleward undercurrent down to the Lüderitz upwelling cell, where it is advected offshore ^17,18^. In consequence, the nBUS is characterized by a permanent, pronounced oxygen-minimum zone (OMZ) below the mixed layer, whereas in the sBUS an OMZ occurs only locally and is seasonally restricted to certain nearshore shelf regions. In the nBUS, the OMZ can determine the vertical distribution of zooplankton and fish based on species- and stage-specific hypoxia tolerance thresholds^19,20^. Vice versa, OMZs can also provide a refuge for hypoxia-tolerant species from predation by more sensitive predators^21,22^.

*Calanoides natalis* (formerly *C. carinatus*) is the dominant upwelling copepod species on the shelf in both the nBUS and the sBUS along the southwestern African coast, while *Calanus agulhensis* prevails on the Agulhas Bank along the south coast of South Africa, a major sardine and anchovy spawning ground^23,24^. So far, the presence of *C. agulhensis* in the nBUS has never been confirmed. Their presumed absence in the nBUS indicates that the westward drift of Agulhas rings and northward transport by the Benguela Jet (also referred to as the Cape Jet) usually do not reach that far north. When upwelling ceases and offshore water masses intrude onto the shelf, the shelf community eventually mixes with species from the open ocean. In the nBUS, the shelf community is then replaced by offshore, “warm-water” species such as *Nannocalanus minor*^16^. Each species is adapted to specific conditions characteristic of their core distribution areas, where their reproductive performance is most successful^23,24^.

*C. natalis* thrives in plumes of nutrient-rich, recently upwelled water close to the coast where it reproduces. A part of the population is transported towards the open ocean by Ekman drift and pre-adult copepodids C5 descend below 200 m. At depth they overcome periods of food shortage in a diapausal state with extremely reduced metabolic activity and extensive lipid reserves^25,26^. At the onset of a new upwelling event they return to coastal surface waters and moult to adults^27,28^. C5s of *C. natalis* usually show a bimodal vertical distribution with maxima at the surface and at depths below 200 m, as only part of the C5 population performs ontogenetic vertical migrations and enters diapause^26,27,29,30^. In contrast, a diapause state has hitherto not been documented for *C. agulhensis*^23,24^.

There have been substantial changes in abundance, biomass, secondary production, species and size composition of neritic zooplankton communities in both the nBUS and the sBUS: copepods have increased since the 1950s in both subsystems, until a turning point around the mid-1990s in the sBUS and about a decade later (after 2005) in the nBUS, when their abundances declined again^1,13,31^. The long-term increase in copepod abundance since the onset of commercial fishing in the 1950s, was accompanied by increasing wind stress and upwelling intensities^32–34^ . In both subsystems, size composition of copepod communities shifted in predominance from larger to smaller species from the 1950s onwards, but data from 2015 onwards are rare^13,31^. A shift from larger to smaller species can be an indicator of ocean warming; however, a cooling trend by up to 0.5 °C per decade was observed from the 1980s onwards due to intensification of upwelling^13,35,36^. The decline of larger copepods such as *C. natalis* in the St Helena Bay region (sBUS) since the mid-1990s coincided with a marked increase of anchovy biomass, potentially having preyed on these larger copepods^1,13,34,37^. In the nBUS, however, the decline of anchovies in the mid-1990s was not followed by an increase of larger copepods^13^. Hence, the relative importance of bottom-up (environmental factors) versus top-down (predation, size-selective feeding) effects remains uncertain^13^.

The present study investigates the abundance, distribution and niche partitioning of Calanidae (copepodite stages C1-5, males, females) in relation to different upwelling intensities and environmental factors during austral summer in the nBUS and sBUS. Calanid species were identified and subsequently grouped into three size classes to examine possible niche partitioning of species competing for similar-sized prey items: the medium-sized species *Mesocalanus tenuicornis* and *N. minor*, the large-sized species *C. natalis* and *C. agulhensis* and the even larger *Neocalanus gracilis* and *N. tonsus*. Fatty acid and stable isotope (δ^15^N) analyses were conducted to identify dietary preferences and trophic positions. We hypothesize (i) that species of the same size class have less distributional or dietary overlap, exhibiting either horizontal, vertical or dietary niche partitioning; (ii) that sympatric distribution despite competition may occur to a certain extent due to continuous recolonization in the highly dynamic BUS and, (iii) that *C. agulhensis* is largely restricted to the sBUS due to the combined effect of the northeastward advection of this species from the Agulhas Bank via the Benguela Jet, and the environmental barrier maintained between the sBUS and nBUS. This is the first study documenting the presence of *C. agulhensis* in this region at mesopelagic depths.

## Results

**Fig. 1:**
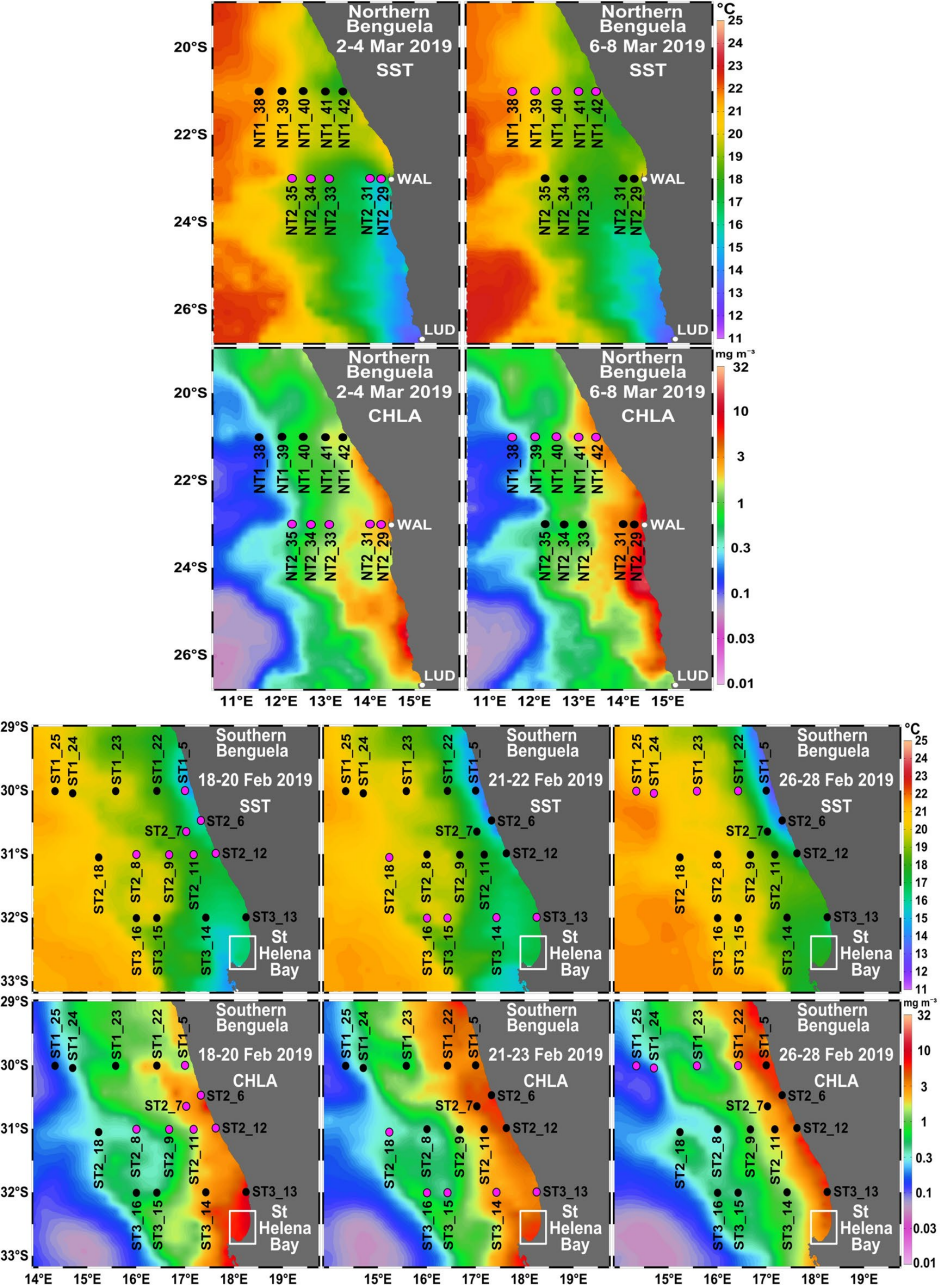
SST and chl *a* (mg m^−3^) superimposed on the transects in the nBUS (NT1, NT2) and the sBUS (ST1, ST2, ST3) during cruise M153. Stations where mesozooplankton was sampled during the respective sampling period are marked in pink. NT, Northern Transect; ST, Southern Transect; WAL, Walvis Bay; LUD, Lüderitz.

The nBUS was characterized by warmer sea surface temperatures (SSTs) compared to the sBUS. In situ SSTs on the nBUS shelf usually ranged between 16.5 and 18.0 °C. SSTs in the sBUS were cooler with minimum values of 12.0 °C at stn. 6, indicating active upwelling, which is also depicted by the satellite data (Fig. 1). Chlorophyll *a* (chl *a*) concentrations were typically higher at the coast and highest, where upwelling cells were observed (stns. 5, 6, 12, 13 in the sBUS and stns. 29 and 31 in the nBUS, see Fig. 1).

**Fig. 2:**
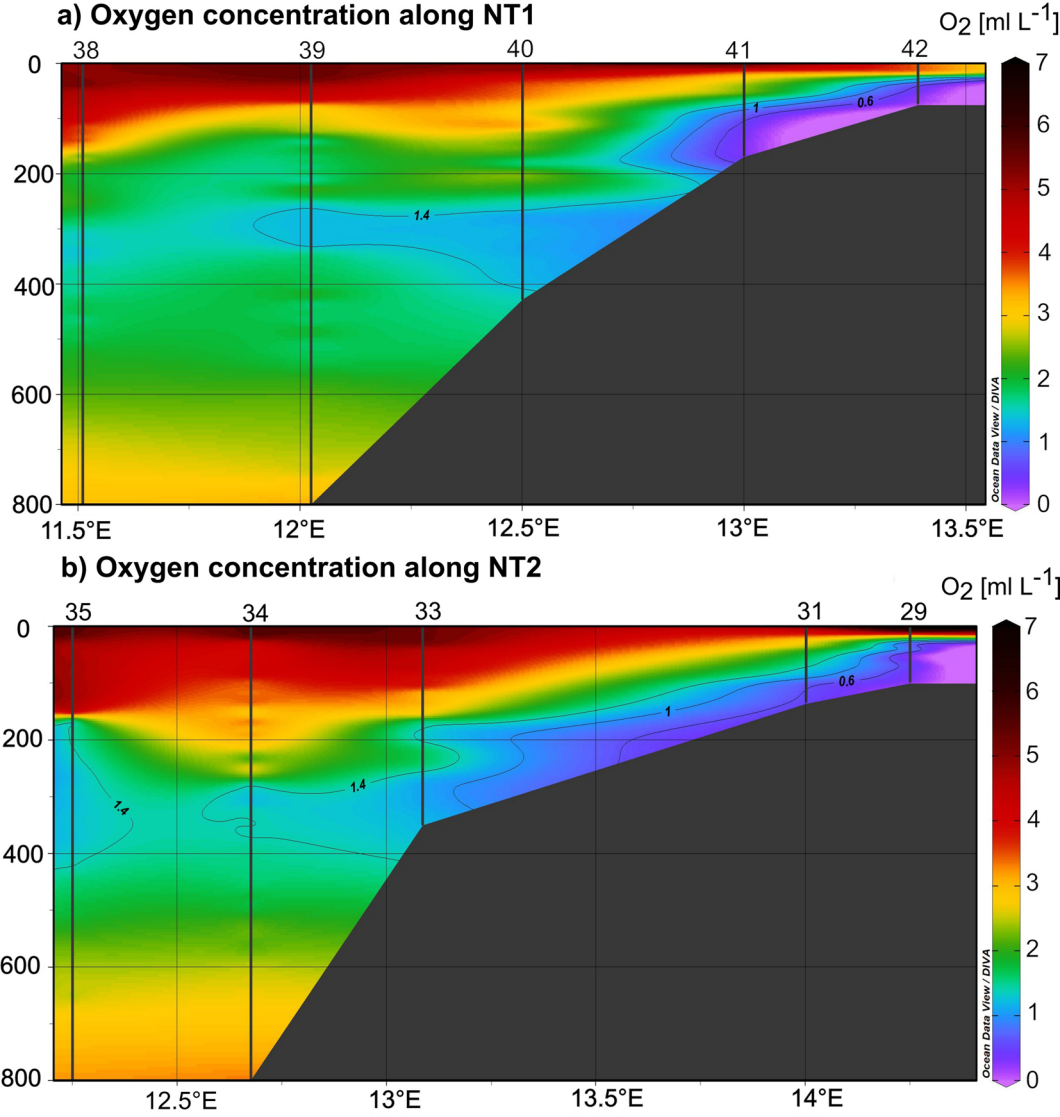
O_2_ profiles (mL O_2_ L^−1^) showing the OMZs along (**a**) NT1 and (**b**) NT2 in the nBUS. Sampled stations are marked with a bold black line. For station locations see Fig. 1. SBUS not shown as pronounced OMZs were absent.

OMZs, defined as depth layers with O_2_ concentrations < 1.4 mL O_2_ L^−1^^20^, occurred along NT1 and NT2 (Fig. 2). Minimum O_2_ concentrations were found inshore at stn. 42 at 48 m depth (NT1) with 0.3 mL O_2_ L^−1^ and at stn. 29 at 54 m depth (NT2) with 0.3 mL O_2_ L^−1^. Along NT1, OMZs occurred at all stations except at the most offshore stn. 38. Closest to the coast, at stns. 42 and 41, the OMZ started at 28 m and 75 m, respectively, with anoxic O_2_ concentrations of < 0.5 mL O_2_ L^−1^ below 48 m and 115 m, respectively, down to the bottom. At stations above the continental slope, hypoxic layers occurred between 270 m and the bottom (410 m) at stn. 40 and between 238 and 281 m at stn. 39. All stations along NT2 were characterized by an OMZ (Fig. 2). In the sBUS along ST3, only two very narrow OMZs were observed inshore at stn. 13 between 32 and 28 m and between 55 and 48 m with minimum O_2_ concentrations of 1.0 mL O_2_ L^−1^.

**Fig. 3:**
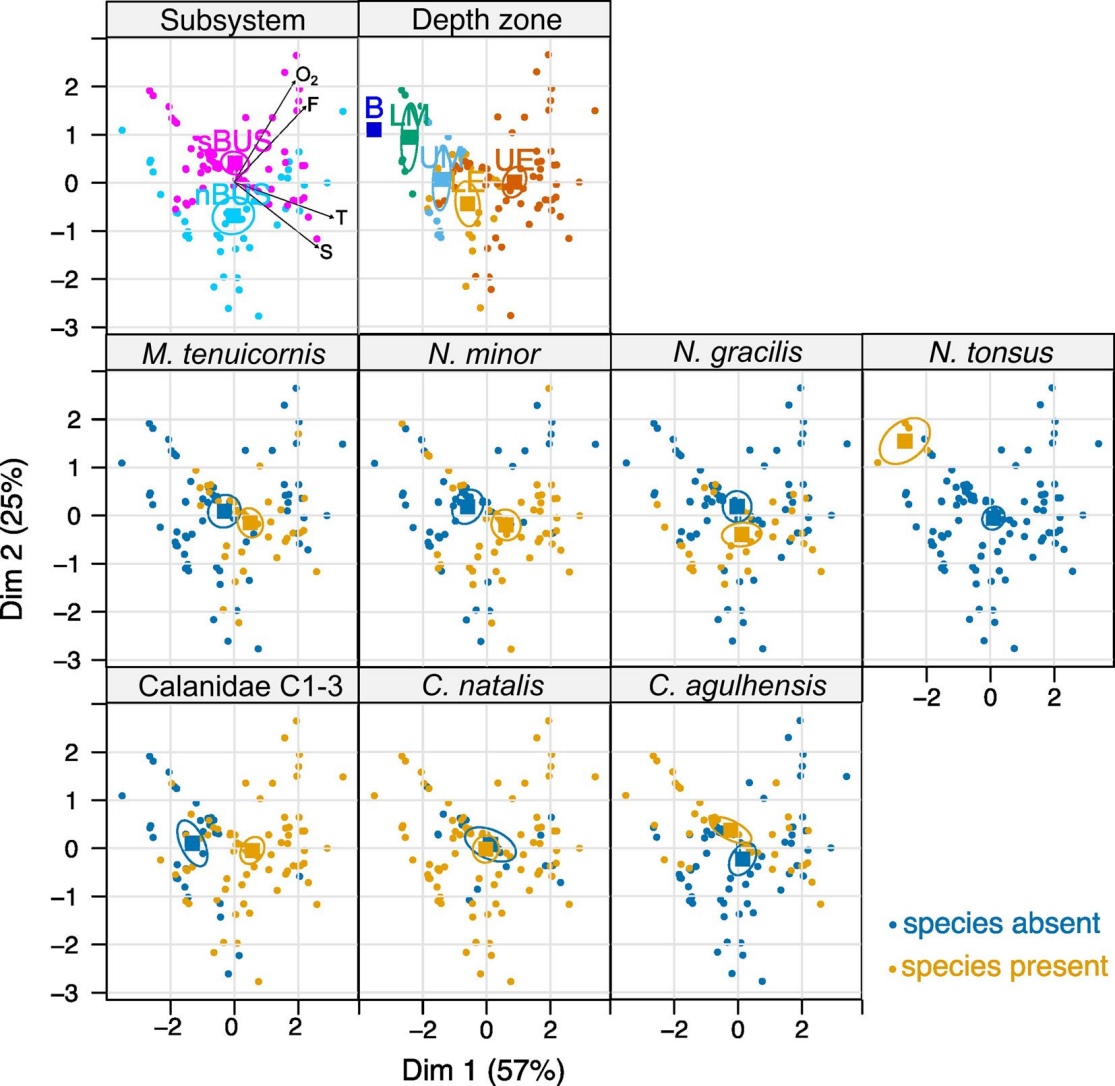
PCA analysis adding supplementary variables as categories: Subsystem (= nBUS, sBUS), depth zone (= upper epipelagic [UE], lower epipelagic [LE], upper mesopelagic [UM], lower mesopelagic [LM], bathypelagic [B]), and presence or absence of respective calanid species (C4-5, females and males) or Calanidae copepodite stages C1-3. The individuals (= sample) are arranged according to the first two principal component dimensions (Dim 1, Dim 2). The environmental variables are indicated as arrows in the upper left figure; O_2_ = oxygen concentration, F = fluorescence signal, T = temperature, S = salinity. Categories of the supplementary variables are each projected at the barycenter (squares) of the individuals (= samples); the barycenter is the average position of all samples belonging to a given category in the ordination space. 95% confidence ellipses were generated around the barycenter of each category.

Principal component analysis (PCA) identified four main components accounting for 99% of variance in the environmental data, determined by temperature, salinity, fluorescence (as proxy for chl *a*) and O_2_ concentration. PC1, explaining 57% of total variance, is mainly represented by temperature, followed by salinity, fluorescence and O_2_ concentration all with positive eigenvectors (*p*-values < 0.0001). PC2, explaining 25% of total variance, showed a significant positive correlation with O_2_ concentration and fluorescence and a significant negative correlation with salinity (*p*-values < 0.0001). In the first categorical dimension, named in order of increasing *p*-values, the depth zones and the presence or absence of Calanidae copepodids C1-3, *N. tonsus*, *N. minor* and *M. tenuicornis* (C4 to adults) had significantly different barycenters (*p*-values ≤ 0.005; Fig. 3), which is the average position of all samples belonging to a given category in the ordination space. In the second categorical dimension, the subsystems, presence or absence of *C. agulhensis*, *N. tonsus*, *N. gracilis* and depth zones had significantly different barycenters (*p*-values ≤ 0.00005; Fig. 3). *C. natalis* was ubiquitously present.

### Overall cross-shelf distribution and species composition

**Fig. 4:**
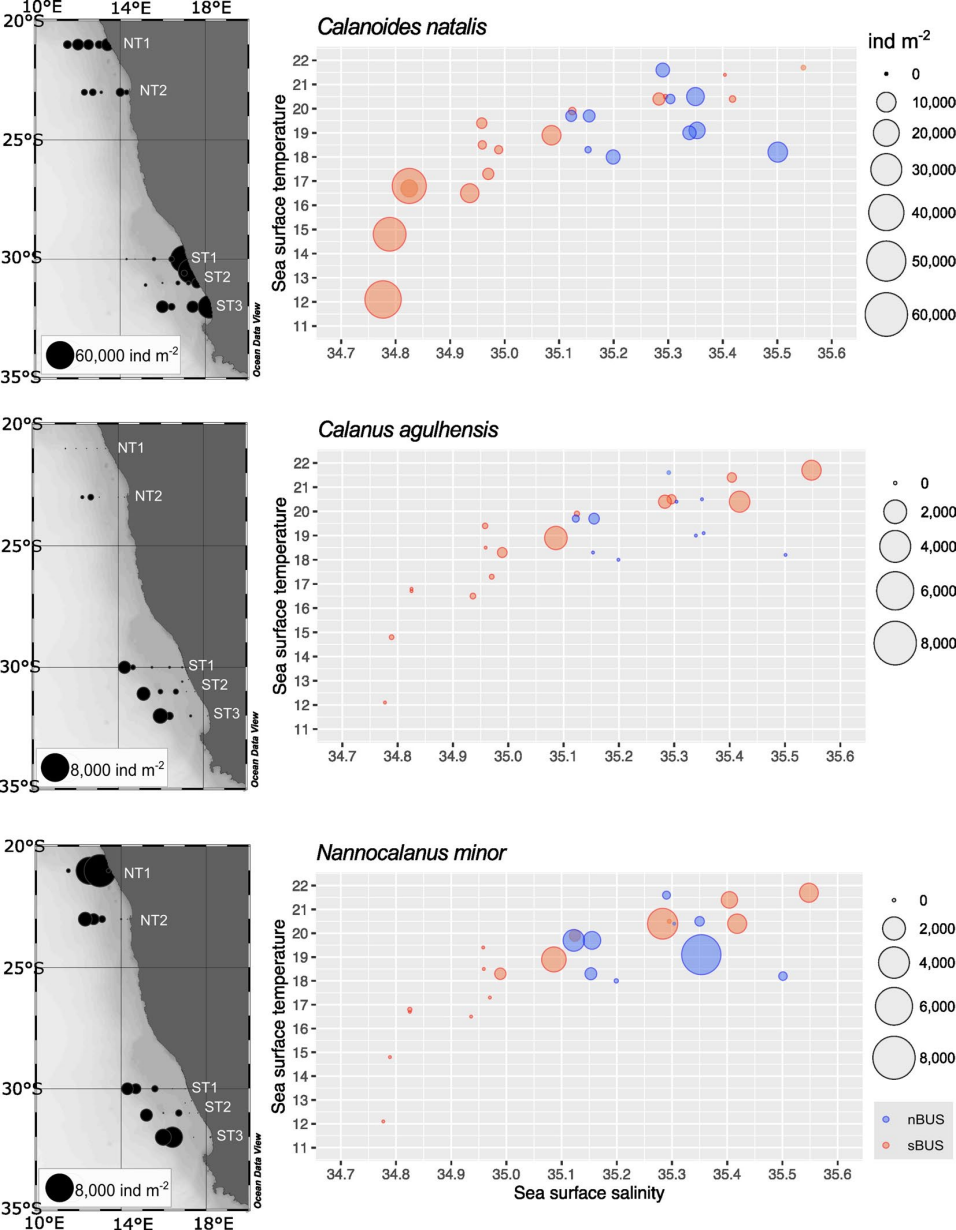
Total abundance (ind m^−2^) of the three most abundant calanid species: *C. natalis*, *C. agulhensis* and *N. minor* (all copepodite stages C4 to C6, including females and males, summed) in the nBUS and sBUS. On the left side, total station abundances are superimposed as sized bubbles over the station positions. On the right, the respective abundance is shown relative to sea surface temperature and sea surface salinity.

**Fig. 5:**
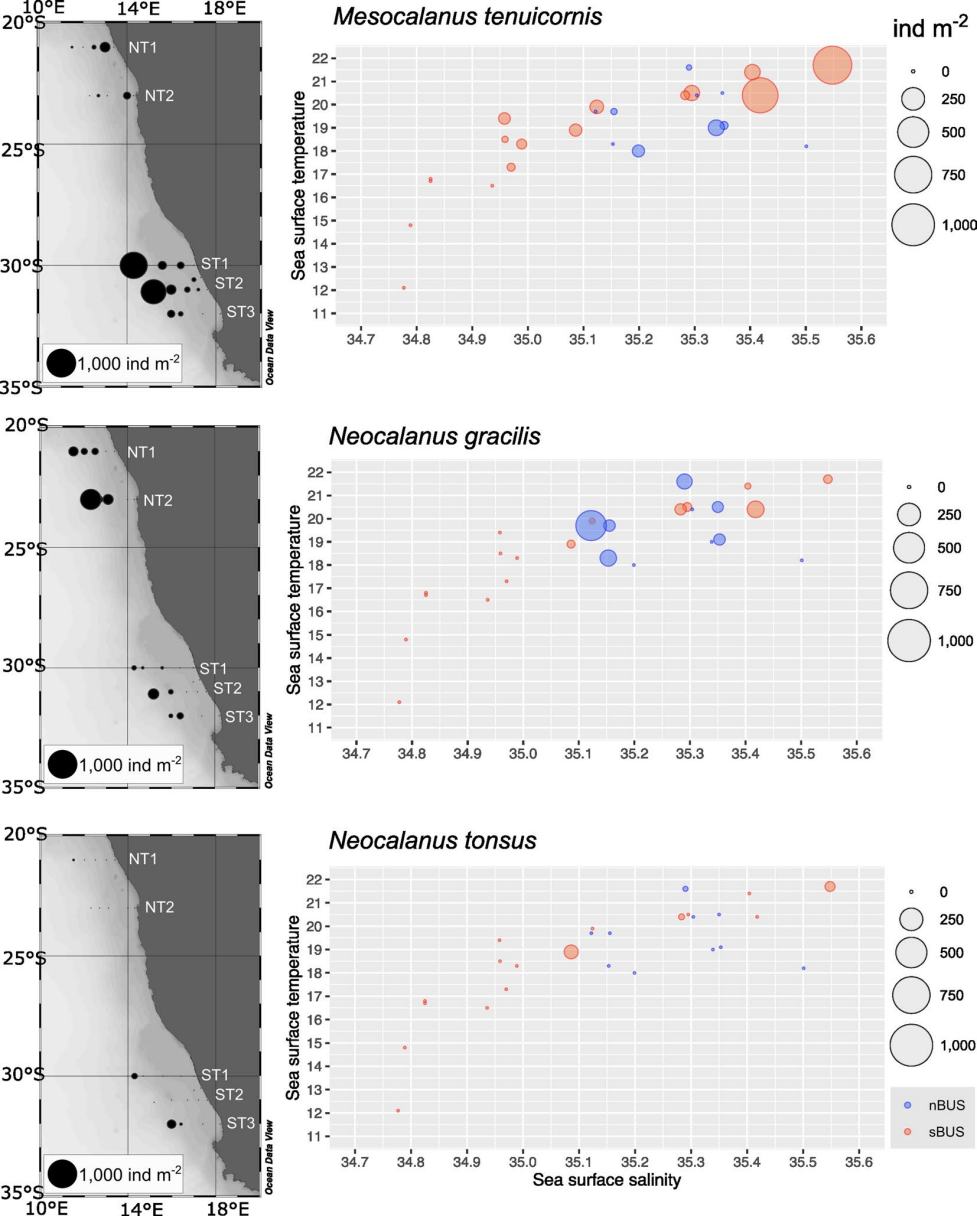
Total abundance (ind m^−2^) of the three less abundant calanid species: *M. tenuicornis*, *N. gracilis* and *N. tonsus* (all copepodite stages C4 to C6, including females and males, summed) in the nBUS and sBUS. On the left side, total station abundances are superimposed as sized bubbles over the station positions. On the right, the respective abundance is shown relative to sea surface temperature and sea surface salinity.

In total, six species of Calanidae were found within the study area. All species occurred in both subsystems and *C. natalis* and *N. minor* were the most abundant species, considering copepodids C4-5, females and males (Figs. 3, 4, Table 1). *C. natalis* usually had highest abundances on the shelf (max. 11,032 ind m^−2^ at the inshore stn. 42 (NT1) in the nBUS, max. 54,414 ind m^−2^ at the inshore stn. 5 (ST1) in the sBUS). In contrast, abundances of *C. agulhensis* increased towards the open ocean with max. 2060 ind m^−2^ (stn. 16, ST3) and were very low in the nBUS with max. 310 ind m^−2^ at the offshore stn. 34 (NT2) (Table 1, Figs. 3, 4). *N. minor* had highest abundances on the shelf with 9981 ind m^−2^ at stn. 41 (NT1). Along all other transects, abundances of *N. minor* increased towards the offshore region with highest abundances of 3973 ind m^−2^ at stn. 15 (ST3). *N. tonsus* only occurred along NT1, ST2 and ST3 with very low abundances (Fig. 5). Early copepodids C1-3 of Calanidae were found at all stations (Table 1). In the nBUS, Calanidae C1-3 occurred usually on the shelf or near the continental slope with abundances between about 2000 and 5000 ind m^−2^. The highest abundance of C1-3 in the nBUS was found offshore along NT2 with 9218 ind m^−2^ at stn. 35. In the sBUS, numbers of Calanidae C1-3 were highest on the shelf with max. 78,004 ind m^−2^ at stn. 5 (ST1). Except for *N. minor* and *N. gracilis*, all species were generally more abundant in the sBUS than in the nBUS (Table 1).

**Table 1:** Total abundance of C6 females (F), C6 males (M), copepodids C4-5 and all copepodite stages C4-6, including females and males, summed (Total) of *C. natalis*, *C. agulhensis* and *N. minor* at each station (ind m^−2^) in the nBUS and sBUS.

			Abundance (ind m^−2^)											
			*Calanoides natalis*				*Calanus agulhensis*				*Nannocalanus minor*			
Loc	Stn	B [m]	F	M	C4-5	Total	F	M	C4-5	Total	F	M	C4-5	Total
NT1	42	76	7624	1109	2298	11,032	−	−	−	−	15	10	144	169
	41	170	1811	719	1770	4300	−	−	−	−	2649	4433	2899	9981
	40	429	482	241	6311	7034	−	−	−	−	1408	598	5033	7038
	39	992	−	8	8657	8665	−	−	−	−	67	42	131	235
	38	> 2000	9	43	4479	4531	−	−	4	4	82	23	29	133
NT2	29	101	312	14	1285	1611	−	−	−	−	−	−	−	−
	31	137	1184	931	2683	4797	−	−	−	−	−	11	−	11
	33	351	101	54	307	463	−	−	−	−	161	8	260	430
	34	1225	36	23	3116	3175	4	−	310	314	273	69	806	1148
	35	> 2000	69	−	2480	2549	−	−	89	89	365	81	1366	1812
ST1	5	128	7387	1755	45,273	54,414	9	−	9	17	−	−	−	−
	22	191	620	146	1444	2211	9	−	8	34	−	−	−	−
	23	208	167	121	521	809	8	−	23	31	83	98	196	376
	24	500	−	−	−	−	7	7	203	217	242	93	610	960
	25	1128	8	−	138	146	−	−	1431	1431	477	545	320	1343
ST2	12	57	5655	80	1890	7625	−	−	−	−	−	−	−	−
	6	61	5918	2193	36,160	44,272	−	−	−	−	−	−	−	−
	7	170	363	55	2272	2690	−	10	10	20	−	−	−	−
	11	196	714	26	400	1141	−	−	−	−	−	−	−	−
	9	254	223	80	804	1106	19	6.3	223	248	121	62	196	380
	8	321	16	17	58	90	61	31	107	200	−	−	8	8
	18	1290	74	66	368	508	341	93	1209	1643	734	227	438	1399
ST3	13	59	28,445	1127	9063	38,635	−	−	−	−	−	−	10	10
	14	161	635	301	8600	9535	7	7	28	41	−	−	−	−
	15	403	703	404	2168	3275	60	−	460	520	1493	304	2177	3973
	16	840	240	530	9489	10,258	42	−	2018	2060	885	353	1251	2489

Minus ( −) indicates absence at the respective station.

Loc, Transect, B, Bottom depth.

### Developmental stage distribution of dominant species

**Fig. 6:**
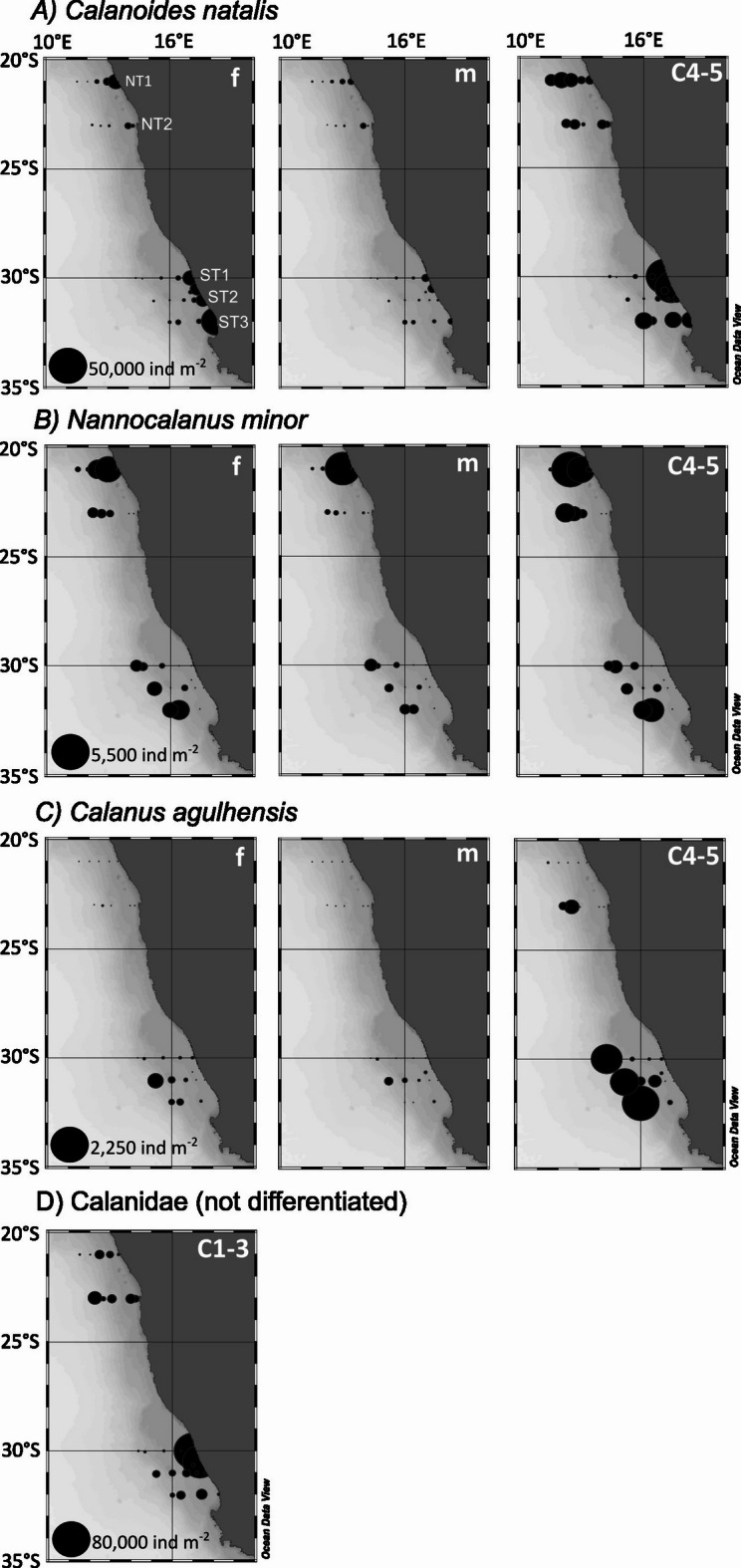
Spatial distribution and total abundance (ind m^−2^) of adult females (f), adult males (m) and copepodids C4-5 of (**a**) *C. natalis*, (**b**) *N. minor* and (**c**) *C. agulhensis* as well as (**d**) copepodids C1-3 of all Calanidae species in the nBUS and sBUS. Note the different bubble scales due to the large variation in abundance.

The three dominant species *C. natalis*, *C. agulhensis* and *N. minor* had distinct distribution patterns in relation to distance from shore. Shelf regions were usually dominated by *C. natalis* females (max. 28,445 ind m^−2^ at stn. 13, ST3) or its copepodids C4-5 (max. 45,273 ind m^−2^ at stn. 5, ST1). Further offshore, copepodids C4-5 prevailed (max. ~ 9000 ind m^−2^ at stn. 39, NT1, and stn. 16, ST3), as the abundance of females generally decreased with increasing distance from shore along all transects (Fig. 6, Table 1). Males of *C. natalis* were similarly distributed as females, but usually with lower abundances reaching max. 2195 ind m^−2^ on the shelf at stn. 6 (ST2).

*N. minor* was usually absent at the stations closest to the shore except along NT1. Females, males and copepodids C4-5 were rather equally distributed with no clearly dominant stage. Females and males prevailed on the shelf at stn. 41 with 2649 ind m^−2^ and 4433 ind m^−2^, respectively, while C4-5 dominated above the continental slope at stn. 40 with 5033 ind m^−2^ (NT1). The abundance of *C. agulhensis* females, males and copepodids C4-5 increased with increasing distance from the shore, with copepodids C4-5 prevailing with max. 2018 ind m^−2^ offshore at stn. 16 (ST3; Fig. 6, Table 1). In general, adult males of the three dominant species were abundant whenever females were present, and, in some instances, even exceeded the abundance of females, especially for *C. natalis* and *N. minor* (Table 1).

### Spatial partitioning of calanid species

**Fig. 7:**
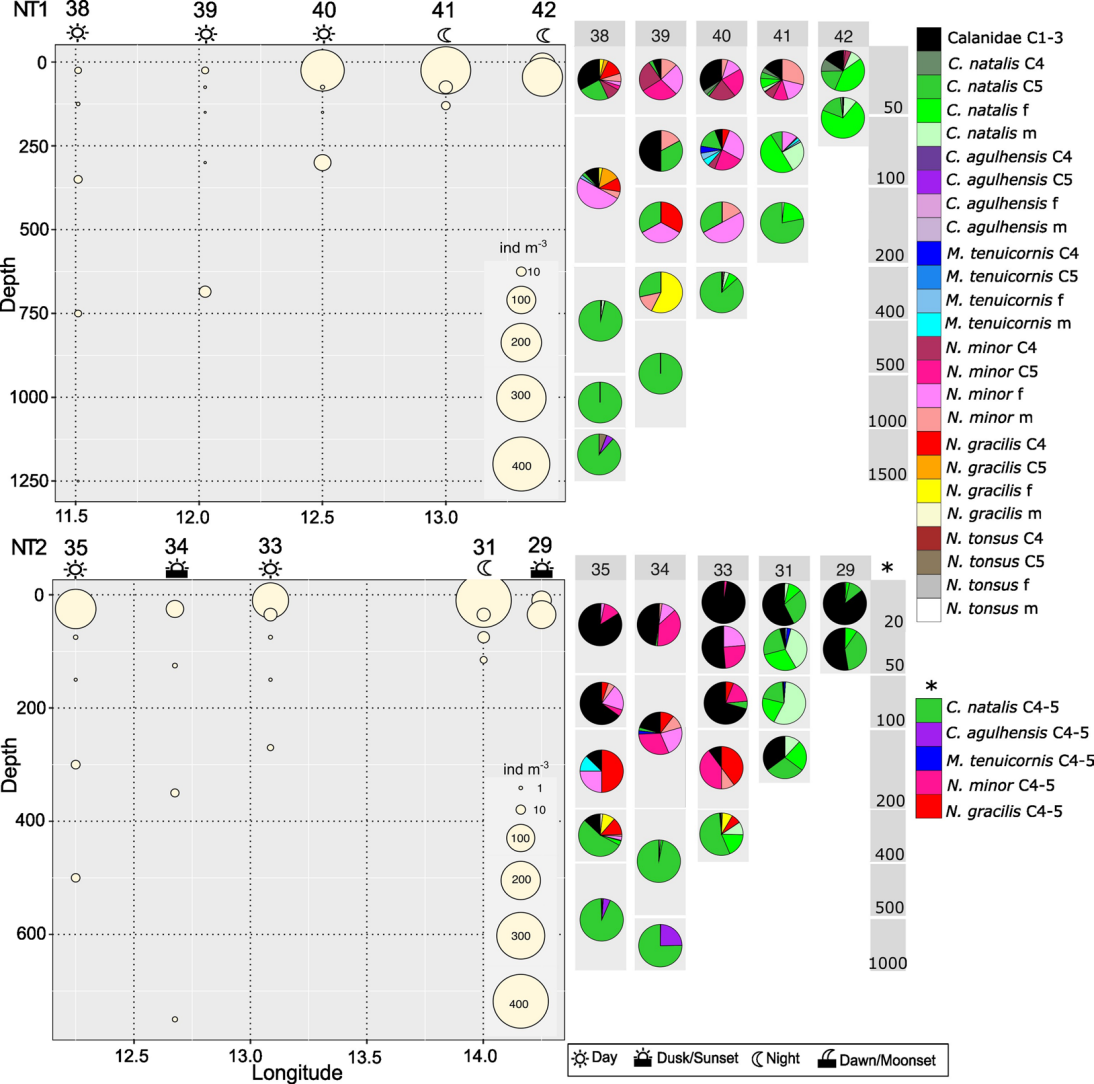
(left) Total calanid abundance (ind m^−3^) per depth layer at stations along NT1 and NT2 with corresponding calanid taxa composition (right). f = female, m = male, C4 = copepodids C4, C5 = copepodids C5. Note that for NT2 (*) copepodids C4-5 were not analyzed separately. Sampling time during the 24 h cycle is indicated by respective sun and moon symbols.

**Fig. 8:**
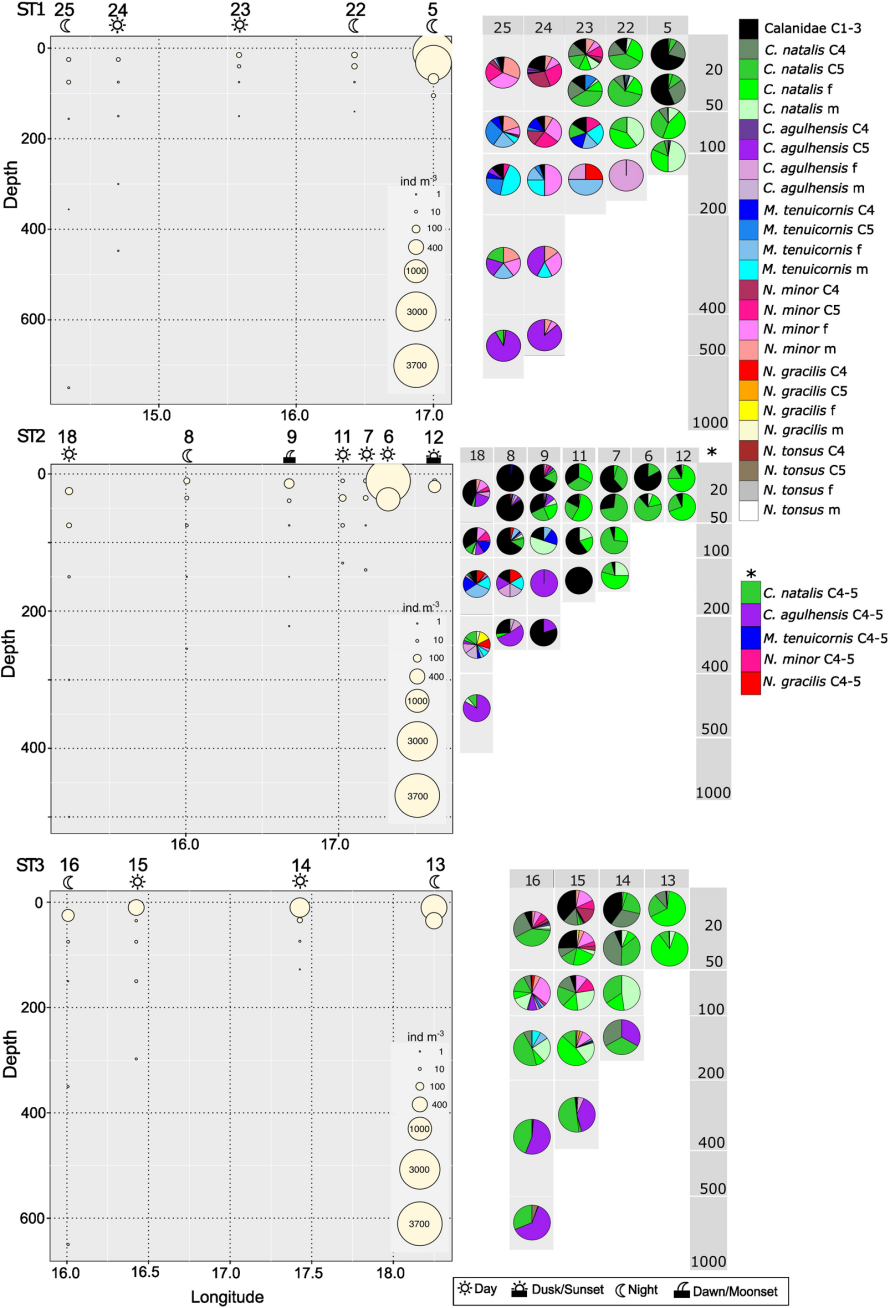
(left) Total calanid abundance per depth layer (ind m^−3^) at stations along ST1, ST2 and ST3 with corresponding species composition (right). f = female, m = male, C4 = copepodids C4, C5 = copepodids C5. Note that for ST2 (*) copepodids C4-5 were not analyzed separately. Sampling time during the 24 h cycle is indicated by respective sun and moon symbols.

Total calanid abundance usually peaked on the shelf. Only along NT2 and ST3 was the overall cross-shelf distribution more uniform with higher abundances also above the continental slope and offshore compared to the other transects, which showed generally low abundances offshore (Figs. 7, 8). Abundances were highest at the surface or in subsurface layers (0–20 m, 20–50 m on the shelf or 0–50 m offshore) and decreased with depth. At several offshore stations a bimodal distribution pattern of total calanids was observed with a smaller secondary abundance peak in the upper mesopelagic (depth layers between 200 and 400 m, i.e. nBUS stns. 34, 35, 38, 39) or lower mesopelagic (depth layers between 400 and 1000 m, i.e. sBUS stns. 25, 16) depth layers. Upper-mesopelagic calanid abundance peaked at stn. 40 (NT1) with 32 ind m^−3^. At lower mesopelagic depths, max. abundance was 5.6 ind m^−3^ at stn. 16 in the 500–800 m depth layer (ST3) (Figs. 7, 8).

#### Northern Benguela

Shelf stations of the northernmost transect NT1 were dominated by older stages of *C. natalis* with a maximum of 139 ind m^−3^ (females), 33 ind m^−3^ (C5) and 19 ind m^−3^ (males) in the 20–50 m depth layer at the inshore stn. 42, equivalent to 71%, 17% and 10% of total calanid abundance (TCA), respectively (Fig. 7; 10.1594/PANGAEA.983970). In the surface layer (0–20 m), early copepodids C1-3 of Calanidae were also abundant with 13 ind m^−3^ (15% TCA). Abundance of adult stages of *C. natalis* in epipelagic layers decreased towards the open ocean. In contrast, *N. minor* were more widespread with maximum abundances of 23 ind m^−3^ (C4), 35 ind m^−3^ (C5), 51 ind m^−3^ (females) and 89 ind m^−3^ (males) on the shelf (0–50 m, stn. 41) and 47 ind m^−3^ (C4), 53 ind m^−3^ (C5), 27 ind m^−3^ (females) and 11 ind m^−3^ (males) above the slope (0–50 m, stn. 40). Below 100 m, particularly in the mesopelagic zone, copepodids C5 of *C. natalis* prevailed peaking between 400 and 970 m at stn. 39, where they comprised 100% TCA (15 ind m^−3^). The layer above (100–200 m) was the only place, where females of *N. gracilis* dominanted (57% TCA) but abundance was < 1 ind m^−3^.

The inshore stn. 29 of NT2 was dominated by early copepodids C1-3 of Calanidae and copepodids C4-5 of *C. natalis* with max. 55 ind m^−3^ (53% TCA) and 40 ind m^−3^ (38% TCA), respectively, at 20–50 m depth (Fig. 7). Abundance along NT2 peaked at stn. 31 in the surface layer (0–20 m): Calanidae C1-3 with 230 ind m^−3^ (57% TCA), *C. natalis* C4-5 with 117 ind m^−3^ (29% TCA), females with 40 ind m^−3^ (10%) and males with 12 ind m^−3^ (3% TCA). Above the continental slope at stn. 33 Calanidae C1-3 (max. at 0–50 m with 164 ind m^−3^, 97% TCA) and, to a lesser extent, C4-5 stages (max. between 20 and 50 m with 5 ind m^−3^, 25% TCA) and females (max. between 20 and 50 m with 5 ind m^−3^, 24% TCA) of *N. minor* were dominant (Fig. 7). Here (stn. 33), *N. minor* copepodids C4-5 had highest contribution to TCA with 40% between 100 and 200 m, but their abundance was low at depth (< 1 ind m^−3^). Calanidae C1-3 and females and copepodids C4-5 of *N. minor* prevailed in the epipelagic zone along NT2 (stns. 34, 35). Below 50 m and particularly below 200 m at the continental slope and beyond, C4-5s of *C. natalis* were dominant, with max. abundances of 8 ind m^−3^ at stn. 35 at 400–600 m (93% TCA) and 7 ind m^−3^ at stn. 34 at 200–500 m (97% TCA).

Copepodids C4-5 of *C. agulhensis* were present along NT2 with low abundances of < 1 ind m^−3^ only very deep, namely 25% TCA between 500 and 1000 m at stn. 34 and 5% TCA between 400 and 600 m at stn. 35 (Fig. 7). Above the continental slope and further offshore along NT2, copepodids C4-5 and females of *N. gracilis* had also larger contributions to TCA in the lower epipelagic (100–200 m) and, to a lesser extent, in the upper mesopelagic zone (200–400 m). *N. gracilis* females occurred with a maximum of 1 ind m^−3^ (13% TCA) between 200 and 400 m at stn. 35, while *N. gracilis* C4-5 were < 1.0 ind m^−3^ (10% TCA). The highest contribution of *N. gracilis* C4-5 with 50% TCA (< 1 ind m^−3^) was detected in the layer above (100–200 m), although abundance in general was low at depth (Fig. 7).

#### Southern Benguela

Similar to the nBUS, inshore stations in the sBUS were dominated by Calanidae C1-3 and copepodids C4-5, females and males of *C. natalis*, but in much higher abundances (Fig. 8, see also Fig. 6, 10.1594/PANGAEA.983970). Highest abundances of Calanidae C1-3 occurred at stns. 6 (ST2) and 5 (ST1) in the surface layer (0–20 m) with 3029 ind m^−3^ (83% TCA) and 2227 ind m^−3^ (70% TCA), respectively. Abundances of C4-5 of *C. natalis* were highest at stn. 5 at 0–20 m (C4: 675 ind m^−3^ (21% TCA), C5: 209 ind m^−3^ (7% TCA) and at 20–50 m (C4: 704 ind m^−3^ (30% TCA), C5: 245 ind m^−3^ (10% TCA)). Maximum abundances of *C. natalis* females were found at stn. 13 (ST3) with 798 ind m^−3^ (0–20 m, 66% TCA) and 416 ind m^−3^ (20–50 m, 85% TCA). Males of *C. natalis* were usually more abundant in subsurface layers (max. 59 ind m^−3^, 6% TCA, at stn. 6 at 20–55 m). Their contributions to TCA were highest at stn. 5 at 90–120 m with 48% (11 ind m^−3^).

TCA above the continental slope and offshore was generally lower than on the shelf and inshore, except for ST3, where TCA was more uniformly distributed across the shelf. Compared to all other slope and offshore stations, TCA along ST3 was high in the uppermost layers above the slope at stn. 15 with 445 ind m^−3^ (0–20 m) and offshore at stn. 16 with 253 ind m^−3^ (0–50 m) (Figs. 6, 8). In addition, highest abundances of Calanidae C1-3 and copepodids C5 of *C. natalis* were observed in the surface layer (0–20 m) at stn. 14 with 280 ind m^−3^ and 165 ind m^−3^, respectively.

Highest abundance of *C. agulhensis* as copepodids C4-5 occurred along ST2 above the slope (stn. 9) with 6 ind m^−3^ at the surface (0–30 m), contributing only 4% to TCA given the high abundances of Calanidae C1-3 and C4-5 and females of *C. natalis*. ST1 and ST3 were the only transects with females and copepodids C5 of *C. agulhensis* above the shelf with higher contributions to TCA but generally low abundances in deeper layers (Fig. 8). The largest contribution of C4-5 *C. agulhensis* to TCA was found at depth with 53% above the continental slope at stn. 8 (ST2, 200–310 m), and with 54% and 63% at stn. 16 (ST3) at 200–500 m and 500–800 m, respectively. Likewise, the largest contribution of adult female *C. agulhensis* to TCA occurred at depth with 17% and 11% also at stn. 8 (ST2, 100–200 m and 200–310 m, resp.), and with 12% offshore at stn. 18 (ST2) at 200–400 m. Their respective abundance at these locations ranged from 1 to 4 ind m^−3^.

Along ST1, *M. tenuicornis* and *N. minor* were widespread from stn. 23 on the shelf and further offshore. *N. minor* occurred at maximum abundance above the slope at stn. 15 (ST3) with C4 (67 ind m^−3^), C5 (38 ind m^−3^), females (66 ind m^−3^) and males (14 ind m^−3^) in 0–20 m. *M. tenuicornis* was usually present with < 1 ind m^−3^. Highest abundances of *M. tenuicornis*, including C4 (2 ind m^−3^), C5 (6 ind m^−3^), females (4 ind m^−3^) and males (1 ind m^−3^), were detected offshore at a depth of 50–100 m at stn. 25 (ST1).

*N. gracilis* occurred almost exclusively above the slope or offshore, except at stn. 23 (ST1), where it was present at 50–100 m on the shelf (25% TCA). Abundances of *N. gracilis* and *N. tonsus* never exceeded 1.0 ind m^−3^.

### Abundance in relation to environmental variables

**Fig. 9:**
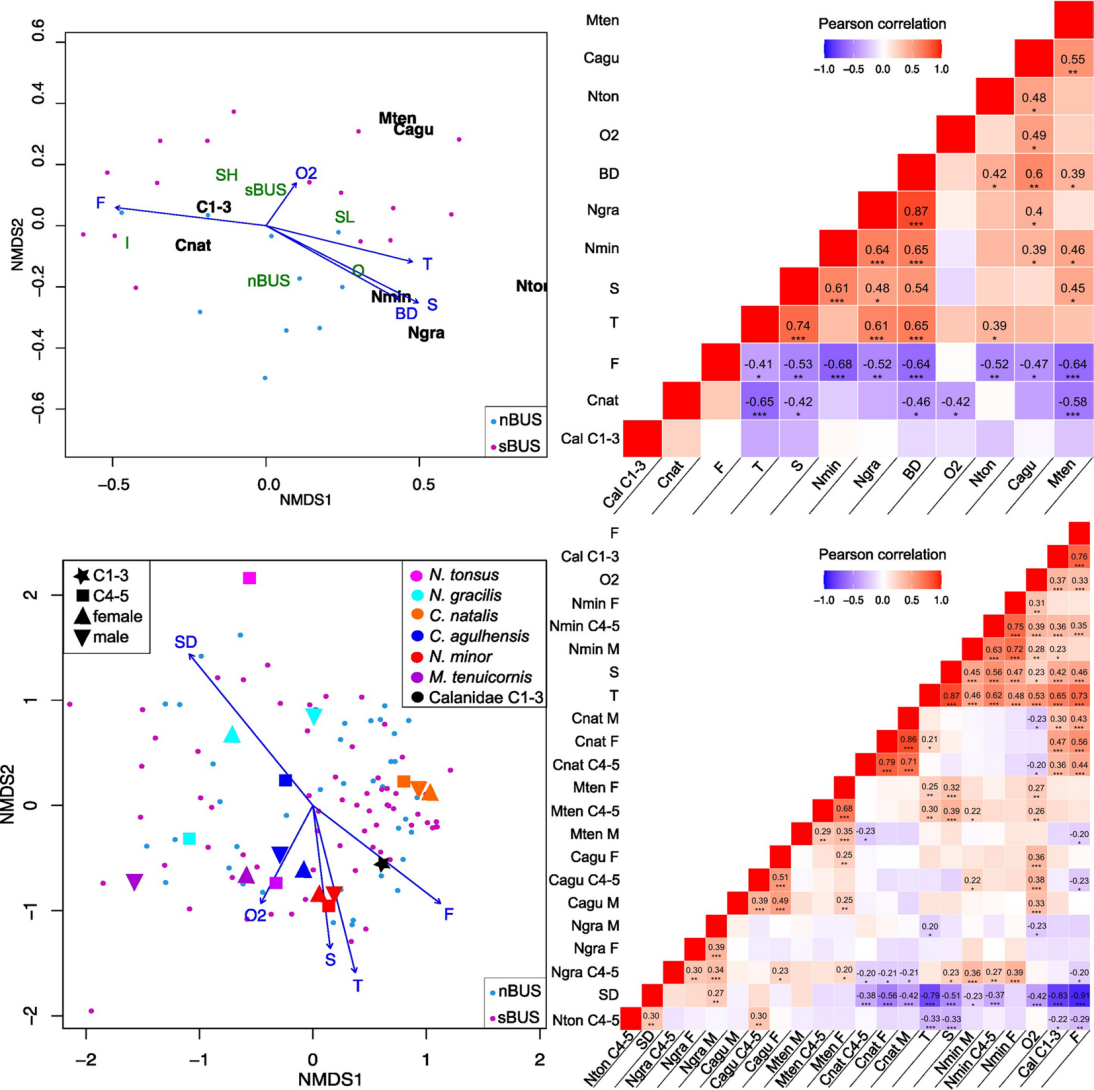
Upper left: nMDS plot of station total calanid abundance (incl. all copepodite stages C1-6) superimposed by environmental variables, i.e. surface temperature (T), salinity (S), fluorescence (F), O_2_ concentration and bottom depth (BD) of the respective station. Upper right: Corresponding Pearson correlation values with significance levels indicated. Lower left: nMDS plot of depth-specific abundances of separated species and copepodite stages, i.e. C4-5, females (F) and males (M) of the respective species (Cnat = *C. natalis*, Cagu = *C. agulhensis*, Nmin = *N. minor*, Mten = *M. tenuicornis*, Ngra = *N. gracilis*, Nton = *N. tonsus*) superimposed by environmental variables, i.e. means of temperature (T), salinity (S), fluorescence (F) and O_2_ concentration of the respective sampling depth interval (SD). Lower right: Significance levels of the corresponding Pearson correlation coefficients, marked by asterisks as *0.05, **0.01, ***0.001.

The sampling area is divided into inshore, shelf, slope and offshore stations as well as nBUS and sBUS based on temperature, salinity, dissolved O_2_ concentration and fluorescence (as proxy for chl *a*) in the surface layer using non-metric multidimensional scaling (nMDS) (Fig. 9, Pearson correlation coefficients with corresponding significance levels). Total abundance of *C. natalis* was significantly negatively correlated with temperature, salinity, O_2_ concentration and depth as dominant factors in both subsystems (Fig. 9). *C. natalis* was associated with higher surface chl *a* concentrations on the shelf, but without significant correlation. *N. minor*, *N. gracilis* and *N. tonsus* showed a significant negative correlation with chl *a* and significant positive correlations with surface salinity and bottom depth towards the nBUS offshore. *C. agulhensis* and *M. tenuicornis* were significantly negatively correlated with chl *a* and were mainly found along the sBUS slope and in the offshore environment where O_2_ concentrations were high (Fig. 9).

In terms of depth- and stage-specific abundances similar patterns were observed, but neither nBUS nor sBUS were a significant factor (Fig. 9). *N. tonsus* and *N. gracilis* were significantly positively correlated with sampling depth (*N. tonsus* C5, *N. gracilis* males *p*-values ≤ 0.01). *C. natalis* had significant negative correlations with sampling depth and O_2_ concentration and a significant positive correlation with chl *a*. In contrast, *C. agulhensis* showed a significant positive correlation with O_2_ concentration, similar to *M. tenuicornis*. Copepodids C4-5 of *C. agulhensis* were more abundant in deeper layers. *N. minor* was significantly positively correlated with temperature, salinity, O_2_ concentration and, in terms of C4-5, with chl *a* (Fig. 9).

### Weighted mean depth

Copepodite stages C1-3 of Calanidae and all stages of *M. tenuicornis* and *N. minor* occurred almost exclusively in the epipelagic zone (0–200 m; Table 2). *C. natalis*, *C. agulhensis* and *N. gracilis* also inhabited the mesopelagic zone (200–1000 m). *N. tonsus* occurred exclusively in meso- and bathypelagic zones (Table 2).

**Table 2:** Weighted mean depth (WMD ± standard deviation [m]) of each taxon and copepodite stage for inshore (IN), shelf (SH), slope (SL) and offshore (OFF) stations across all transects.

Taxon	Stage	PL (mm)	IN (m)	SH (m)	SL (m)	OFF (m)
Calanidae	C1-3	0.8 ± 0.2 (37)	15 ± 7 (5)	29 ± 27 (8)	22 ± 9 (6)	41 ± 13 (7)
*C. natalis*	C4	–	11/28 (2)	21 ± 5 (5)	19 ± 8 (3)	50 ± 35 (4)
	C5	1.9 ± 0.1 (21)	15/40 (2)	32 ± 17 (5)	167/289 (2)	521 ± 286 (4)
	F	2.1 ± 0.3 (7)	29 ± 11 (5)	37 ± 23 (8)	173 ± 112 (5)	186 ± 154 (6)
	M	1.9 ± 0.1 (7)	27 ± 10 (5)	53 ± 34 (8)	158 ± 114 (5)	309 ± 251 (5)
*C. agulhensis*	C4	–	Absent	10/15 (2)	25/28 (2)	26 (1)
	C5	1.8 ± 0.1 (6)	Absent	53 ± 37 (4)	248/339 (2)	829 ± 382 (3)
	F	2.2 ± 0.1 (16)	Absent	101 ± 63 (4)	92 ± 95 (4)	279 ± 407 (3)
	M	2.1 ± 0.1 (4)	Absent	10/35 (2)	61 ± 51 (3)	144 (1)
*M. tenuicornis*	C4	–	Absent	25/75 (2)	75/75 (2)	25/77 (2)
	C5	–	Absent	31 ± 7 (3)	25/150 (2)	57/80 (2)
	F	1.7 ± 0.0 (4)	Absent	51 ± 37 (6)	98 ± 47 (5)	106 ± 31 (4)
	M	1.5 ± 0.1 (8)	Absent	53 ± 37 (3)	75/150 (2)	150/174 (2)
*N. minor*	C4	–	10/16 (2)	23/25 (2)	36 ± 33 (3)	30 ± 6 (4)
	C5	–	33 (1)	18/25 (2)	15/25 (2)	27 ± 3 (4)
	F	1.6 ± 0.1 (30)	45 (1)	15/27 (2)	43 ± 42(5)	56 ± 29 (7)
	M	1.3 ± 0.1 (15)	10 (1)	16 ± 7 (3)	60 ± 61 (4)	54 ± 23 (5)
*N. gracilis*	C4	1.7 ± 0.2 (3)	Absent	91 (1)	75 (1)	95 ± 43 (4)
	C5	2.3 ± 0.2 (16)	Absent	Absent	115/300 (2)	69 ± 77 (3)
	F	3.0 ± 0.2 (8)	Absent	Absent	94/270 (2)	215 ± 160 (5)
	M	–	Absent	Absent	270/300 (2)	300/350 (2)
*N. tonsus*	C5	2.5 (1)	Absent	Absent	297 (1)	883 ± 321 (3)
	F	2.8 ± 0.2 (6)	Absent	Absent	Absent	Absent

PL = Mean (± standard deviation) measured prosome length (mm). The number in brackets indicates the number of individuals measured or number of observations.

Weighted mean depths (WMDs) differed among copepodite stages and locations. *C. natalis* C5 had shallow WMDs between 15 m and 32 ± 17 m on the shelf and deep WMDs of 521 ± 286 m offshore, but due to their known bimodal distribution calculating WMDs is difficult for this species. *C. agulhensis* C5 were absent inshore, had a WMD of 53 ± 37 m on the shelf and 829 ± 382 m offshore (Table 2). Adult females of both *C. natalis* and C. *agulhensis* also occupied deeper WMDs above the slope and offshore (*C. natalis* 173 ± 112 m and 186 ± 154 m, resp.; *C. agulhensis* 92 ± 95 m and 279 ± 407 m, resp.) with very large variation in their vertical distribution, while their copepodids C4 always remained in upper epipelagic layers. *M. tenuicornis* females showed increasing WMDs towards offshore (to max. 106 ± 31 m), while *N. minor* remained strictly within the upper 100 m layer (max. WMD offshore with 56 ± 29 m). *N. tonsus* C5s had the deepest WMD of 883 ± 321 m offshore.

### Dietary preferences and lipid storage among Calanidae

δ^15^N values of the calanid copepods ranged between 8.0‰ and 10.0‰ (Table 3). Early-life stages tended to have lower δ^15^N values, which indicate a higher contribution of lower trophic levels to their diet. Lowest δ^15^N values were determined in copepodite stages C1-3 of Calanidae (8.2 ± 0.4‰) as well as among adult females of the medium-sized *M. tenuicornis* (8.4 ± 0.1‰) and *N. minor* (8.9 ± 0.2‰). Females and males of *C. natalis* had the highest δ^15^N values with 9.7 ± 0.2‰ and 10.2 ± 0.4‰, respectively.

**Table 3:** Stable nitrogen (δ^15^N_fix_, ‰) isotopic ratios of formalin-fixed calanid species and copepodite stages and summary of fatty acid (FA) results from frozen calanid species and stages.

Taxon	Stage	δ^15^N_fix_	Σ20:1	Σ22:1	ΣDia	CI	BI	TL (%DM)	WE (% TL)	n_FA_
Calanidae	C1-3	8.2 ± 0.4 (3)								
*N. minor*	C4-5	8.3/8.6								
	F	8.9 ± 0.2 (6)	0.3 ± 0.2	0.0 ± 0.0	2.8 ± 2.1	0.4 ± 0.1	0.4 ± 0.1	18.6 ± 10.4	0.6 ± 0.8	9
	M	9.4 ± 0.2 (5)	0.0/0.5	0.1/0.2	0.7/1.0	0.5/0.6	0.6/0.6	5.2/5.2	0.4/0.6	2
*M. tenuicornis*	C4	6.5								
	C5	8.3/8.6								
	F	8.4 ± 0.1 (4)								
	M	9.8								
*C. natalis*	C4	7.8								
	C5	9.6 ± 0.5 (7)	11.0 ± 3.4	14.9 ± 4.7	13.5 ± 1.9	0.2 ± 0.0	1.0 ± 0.0	44.0 ± 9.2	79.0 ± 5.8	17
	F	9.7 ± 0.2 (5)	3.7 ± 2.5	4.7 ± 3.6	8.7 ± 3.8	0.2 ± 0.1	0.1 ± 0.0	20.0 ± 12.7	34.7 ± 21.9	9
	M	10.2 ± 0.4 (5)								
*C. agulhensis*	C4	8.3 ± 0.3 (5)								
	C5	9.0 ± 0.2 (6)	3.1/2.1	4.3/5.7	2.7/1.0	0.1/0.5	0.1/0.5	15.7/19.8	42.2/62.2	2
	F	9.0 ± 0.1 (3)	0.8 ± 0.7	0.3 ± 0.4	2.7 ± 2.6	0.4 ± 0.2	0.4 ± 0.2	12.5 ± 7.2	8.3 ± 8.7	5
	M	9.8 ± 0.4 (3)								
*N. gracilis*	C4	8.9 ± 0.5 (4)								
	C5	9.8 ± 0.3 (5)								
	F		1.3 ± 0.3	8.1 ± 4.8	2.9 ± 0.2	0.5 ± 0.0	0.3 ± 0.0	22.7 ± 18.0	28.1 ± 11.3	5
*N. tonsus*	F		2.7 ± 2.7	2.1 ± 2.3	2.0 ± 0.9	0.5 ± 0.1	0.4 ± 0.1	12.3 ± 3.3	18.1 ± 16.4	8

FA compositions are given as percentages of total FAs and were summed for specific biomarker FAs (Σ20:1 = sum of 20:1(n-11), 20:1(n-9, 20:1(n-7); Σ22:1 = sum of 22:1(n-11), 22:1(n-9), 22:1(n-7); ΣDia = sum of diatom marker FAs 16:1(n-7), 16:4(n-1), 18:1(n-7); CI = carnivory index; BI = bactivory index; TL = total lipid (% of DM); WE = wax ester (%TL). When only two samples were available both measured values are given. When samples size was ≥ 3, means ± standard deviations are shown. Number of replicates are indicated in brackets for δ^15^N_fix_ and as n_FA_ for FA measurements.

**Fig. 10:**
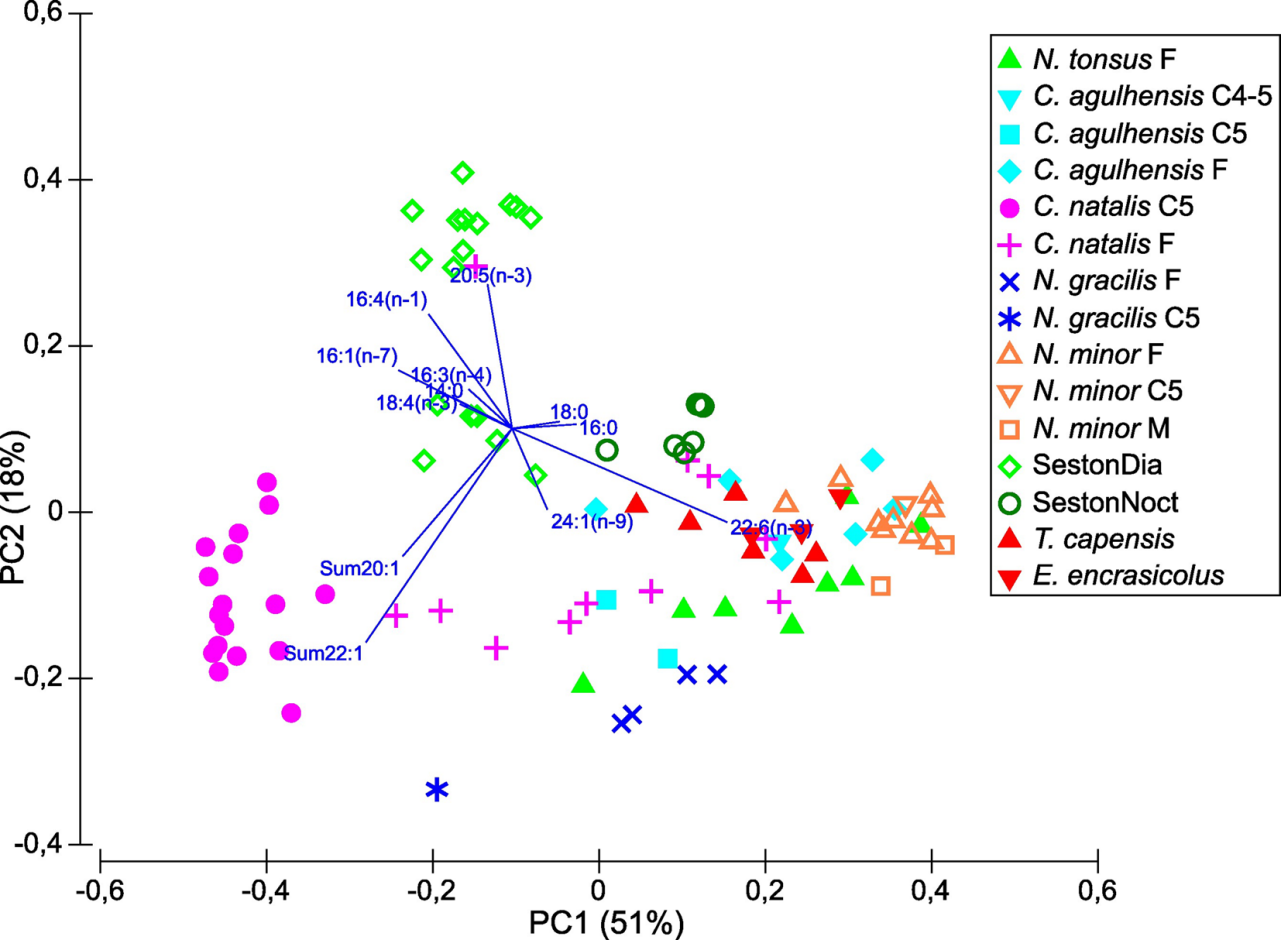
PCA based on fatty acid (FA) compositions. Calanid species and stages (females, males, copepodids C5) are arranged along the first principal components PC1 (explained 51% of variation) and PC2 (explained 18% of variation). For comparative purposes, seston samples containing high amounts of diatoms (SestonDia) and the dinoflagellate *Noctiluca* (SestonNoct) were added in the PCA to illustrate FA compositions of potential copepod prey. Likewise, measurements of larvae of Cape horse mackerel *Trachurus capensis* and anchovy *Engraulis encrasicolus* were added to illustrate FA compositions of potential copepod predators.

Fatty acid (FA) compositions revealed that diatoms prevailed in the diets of copepodids C5 and, to a lesser extent, of females of *C. natalis*. Accordingly, they had the highest amounts of typical diatom trophic biomarkers (Table 3, Fig. 10). Highest amounts of wax esters (WEs) were stored by copepodids C5 of *C. natalis* (79 ± 6% TL) followed by C5s of *C. agulhensis* (42% and 62% TL). Lower and highly variable WE contents were determined in females of *C. natalis* (34.7 ± 21.9% TL), *N. gracilis* (28.1 ± 11.3 TL), *N. tonsus* (18.1 ± 16.4% TL) and *C. agulhensis* (8.3 ± 8.7% TL) (Table 3).

Three main principal components (PCs) were identified explaining 80% of the variance in the FA composition, with PC1 and PC2 together explaining 69% (Fig. 10). The first PC was mainly represented by positive eigenvectors of the FAs 22:6(n-3), 16:0, 18:0, 24:1(n-9) and negative eigenvectors of the sums of 22:1 and 20:1 monounsaturated FAs (MUFAs), respectively, 16:1(n-7), 16:4(n-1), 14:0, 18:4(n-3) and 16:3(n-4), mentioned with decreasing explanatory power (overall PC1 explained 51% of variance). The second PC was mostly represented by positive eigenvectors of 20:5(n-1), 16:4(n-1), 16:1(n-7), 16:1(n-7) and negative eigenvectors of the sums of 22:1 and 20:1 FAs, respectively, 22:6(n-3), 24:1(n-9), mentioned with decreasing explanatory power (overall PC2 explained 18% of variance). The seston samples consisting of high amounts of diatom tracers had a distinct cluster containing typical diatom markers 16:1(n-7), 16:4(n-1), 20:5(n-3), but also, to a lesser extent, 18:4(n-1) indicating the presence of flagellates in the sample (Fig. 10). The genera *Calanoides* and *Neocalanus* had the highest amounts of the MUFAs 22:1(n-11) and 20:1(n-9), typical of calanid copepods and represented as sums of 22:1(n-11, n-9, n-3) and 20:1(n-11, n-9, n-3), respectively, in Fig. 10. Especially copepodids C5 of *C. natalis* had high amounts of 20:1(n-9), which is usually a major component of WEs (storage lipid), and higher amounts of diatom markers, clustering separately from the other calanids. Except for two samples, they were deep diapausing C5s of *C. natalis*. Potential predators such as larval stages (2–5 mm) of anchovy *Engraulis encrasicolus* and Cape horse mackerel *Trachurus capensis* clustered with the large calanid cluster, potentially preying on nauplii and earlier copepodite stages, thus reflecting their FA biomarker signature. This large cluster was characterized by higher amounts of 22:6(n-3) and lower amounts of diatom FA markers, indicating a more mixed diet.

## Discussion

The present study highlights the presence of fine-scale niche partitioning of closely related, similar-sized calanids in the pelagic realm, promoting co-occurrence and diversity also in highly dynamic coastal upwelling systems. Calanidae could be separated into three size categories each with potentially similar functional traits such as prey size spectra^23^. The medium-sized species *M. tenuicornis* and *N. minor* (adult stages 1.3–1.7 mm prosome length (PL), Table 2), the large-sized *C. natalis* and *C. agulhensis* (adult stages 1.9–2.2 mm PL) and the very large *N. gracilis* and *N. tonsus* (adult stages 2.8–3.0 mm PL) were distinguished. We demonstrated that closely related species within the same size class exhibited horizontal, vertical or dietary niche partitioning. However, these differences are not easily discerned as distribution overlap also occurred. Continuous recolonization in the highly dynamic BUS likely promotes sympatric distribution despite competition and may be stronger than in other non-coastal upwelling regions. This mechanism is likely even more pronounced in the sBUS than in the nBUS due to the direct link with the Agulhas Current and the Southern Ocean via the Subantarctic Mode Water (SAMW)^38^. Thus, we can accept our first two hypotheses, namely (i) that species of the same size class have less distributional or dietary overlap, exhibiting either horizontal, vertical or dietary niche partitioning, and (ii) that sympatric distribution despite competition may occur to a certain extent due to continuous recolonization in the highly dynamic BUS.

We can only partially accept the third hypothesis, that *C. agulhensis* is restricted to the sBUS, due to a combined effect of its northeastward advection from the Agulhas Bank via the Benguela Jet and the environmental barrier between the sBUS and nBUS. Unexpectedly, *C. agulhensis* occurred sporadically on both nBUS transects offshore, confirming that the Lüderitz upwelling cell is not a strict environmental barrier for this species^39^. As they were mainly found at mesopelagic depth in the nBUS, the species was most likely transported into the nBUS via aged Agulhas retroflection rings drifting towards the nBUS.

Adult males of *C. natalis* and *N. minor* were generally fairly abundant whenever females were present. At some sites, males were even exceeding the abundance of females, which suggests active reproduction in both subsystems at the time of sampling. Adult males of *C. agulhensis* were also present together with females, especially in the southern part of the sBUS, but their reproductive success seems limited in that area, as this species could not establish a high population size on the shelf. Overall, *C. natalis* was the dominant species. The early copepodids C1-3 of unidentified Calanidae were most abundant in regions affected by upwelling in the sBUS and we assume therefore that the majority of these C1-3 specimens on the shelf were most likely *C. natalis*.

All calanid species, except for *N. minor* and *N. gracilis*, were on average less abundant in the nBUS than in the sBUS. Contrasting upwelling seasonality in the two subsystems may explain these differences. While there was active upwelling in the sBUS during the sampling period (February/March), there is usually perennial upwelling in the nBUS and high copepod abundances often occur in December and March on the shelf with abundances of *C. natalis* between 20,000 and 180,000 ind m^−2^ during peak seasons^16^. Maximum abundances of *C. natalis* in the present study were 11,032 ind m^−2^ in the nBUS and 54,414 ind m^−2^ in the sBUS. To identify seasonal or long-term effects, time-series data are needed, which was not within the scope of this study. Yet, copepod abundances fell within the range of previously reported interannual variability^15,16,26,40,41^.

### Distribution and niche partitioning of species with similar functional traits

Calanid copepods are current-feeding herbivores-omnivores^42,43^, consuming both phytoplankton- and microzooplankton-based food items, and occupying similar trophic positions, which was reflected by their similar δ^15^N values. The two biomass-rich key species, *C. natalis* and *C. agulhensis*, share common functional traits, i.e. they are about equal in size and display similar feeding modes^23^. Yet, *C. natalis* appears to feed more size-selectively, as particle size was more important for this species in previous studies, i.e. preferring diatoms over flagellates^24^. This particle selectivity may not only be based on size but also on quality. *C. natalis* egg production rates increased when the proportion of large phytoplankton cells (> 10 µm) exceeded 50% of their diet^24^. This was reflected in the present study by a higher portion of diatom trophic marker FAs in *C. natalis* compared to *C. agulhensis*. On the Agulhas Bank, *C. agulhensis* was observed to feed on a mixture of diatoms and flagellates^44^. In addition to differences in their dietary niche, the two species showed spatial niche separation: *C. natalis* was generally associated with colder, phytoplankton-rich shelf waters, whereas *C. agulhensis* were predominantly found in warmer offshore waters in the sBUS due to the different dispersal patterns of their associated water masses^30^. Older copepodite and adult stages of *C. agulhensis* are advected to the South African west coast in the sBUS, where its abundance is associated with warmer offshore waters. However, *C. agulhensis* is also found in the productive cold ridge of upwelled water on the central and eastern Agulhas shelf^23^. Even though their distribution overlapped, abundance of the upwelling specialist *C. natalis* in the sBUS and on the west coast shelf usually exceeds that of *C. agulhensis*, whereas *C. agulhensis* on the Agulhas Bank is usually more abundant than *C. natalis*^23,24,44^.

Both, *N. minor* and *M. tenuicornis* were most abundant above the continental slope and offshore, associated with highest SSTs in the sBUS. They only occurred in low abundances on the shelf. In contrast, in the nBUS both species were most abundant in waters on the shelf, associated with comparatively high SSTs and thus likely influenced by southward warm-water intrusions of Angola Current water onto the shelf^16^. In agreement with our results, a preference for warmer water masses has been reported for both species^16,45^. *N. minor* was comparatively more abundant in the nBUS than in the sBUS, supporting earlier observations that *N. minor* belongs to the dominant copepod species in the nBUS^16,46^ but not in the sBUS^28,47^. *M. tenuicornis*, in comparison with *N. minor*, occurred at highest abundances at the very offshore stations in the sBUS; its distribution pattern has not been studied before in the BUS. In the California upwelling region, *M. tenuicornis* was exclusively found offshore^45^. Vertical preferences of developmental stages of *N. minor* could not be distinguished^48^, indicating the absence of ontogenetic vertical migration for this species and likely also for *M. tenuicornis*. Developmental stages dwelling at similar depths are therefore equally affected by advection and other water movements caused by upwelling. However, *M. tenuicornis* was generally exhibiting deeper WMDs than *N. minor*, thereby reducing vertical overlap and indicating fine-scale differences in vertical niche partitioning. In addition, *M. tenuicornis* is slightly larger in size and has a strikingly large maxilliped (see drawings in^49,50^), indicative of different dietary spectra and thus trophic niches.

Not much is known about the distribution of the less abundant species *N. gracilis* and *N. tonsus* in the BUS. Both species have similar distribution patterns in the two subsystems and their abundances were equally low so that competition between the two species was unlikely in the present study. *N. gracilis* scattered from the surface to mesopelagic depths with higher abundances in the nBUS compared to the sBUS. *N. tonsus* occurred as deeper-dwelling (> 200 m) copepodids C5 above the slope and offshore and were therefore not affected by movements of surface currents. Elsewhere, both species have been associated with warmer water masses in the Pacific Ocean^51^ and in the Scotia Sea in the South Atlantic^52^. *N. tonsus* mainly thrives at subantarctic to subtropical latitudes of the Southern Hemisphere and it is also known to occur within the sBUS ^53–55^, but occurrences of this species in the nBUS have not been reported to our knowledge. *N. tonsus* can be associated with the northward drift of subantarctic waters^56^ and may indicate the presence of these water masses in the study area. *N. tonsus* was possibly carried into the BUS with SAMW and was then transported further north^38^. As most copepodids C5 of *N. tonsus* enter diapause at depth^53^, these mesopelagic C5s were likely in diapause. C5s of both *N. gracilis* and *N. tonsus* were not sampled alive, so lipid composition could only be determined for females, which had moderate but highly variable amounts of WEs (Table 3), likely depending on their reproductive status.

### The special life cycles and ecological roles of *C. natalis* and *C. agulhensis*

The typical spatial distribution pattern of *C. natalis* was observed in accordance with previous studies^24,26,30,41^. Adult stages of *C. natalis* prefer nutrient-rich regions to ensure sufficient energy for egg production and mate finding for fertilization^41^. High abundances of adult males and females in shelf regions of the sBUS, particularly at 30°S and 32°S, likely resulted from previously deep-dwelling, diapausal C5 copepodids that were advected inshore with the upwelling front and subsequently moulted into adults^28,41^. Males of *C. natalis* were generally less abundant than females. Their abundance, when compared to females, was higher beyond the shelf. Females tend to dwell in deeper layers than males during the day and are thereby less affected by advection of the upper water layer than males^55,57^. Female calanid copepods can store the sperm upon copulation and successful spermatophore transfer^58^, allowing longer-term fertilization of their eggs in the absence of males^58,59^. To our knowledge, it is not known how long *C. natalis* can store the sperm. Interestingly, species lacking seminal receptacles and therefore being unable to store sperm have a sex ratio close to 1:1^60^.

The distribution patterns of *C. natalis* fitted well to the observed oceanographic patterns and the different stages of upwelling. Offshore at ST1, the abundance of *C. natalis* C5s below 200 m was low, indicating that only a small fraction of the diapausal C5 population remained offshore at depth and that upwelling was ongoing. SST and chl *a* concentrations in nearshore, newly upwelled water masses are usually low^55^. In contrast, ST3 was characterized by diminished upwelling, indicated by higher SST inshore and high chl *a* concentrations above the continental slope compared to ST1 and ST2, suggesting an offshore advection of phytoplankton and a well-developed shelf and slope population of *C. natalis*. Offshore at NT1 and ST3, abundances of *C. natalis* C5s were particularly high below 200 m. Apparently, they belonged to the diapausal population that can be permanently found between 200 and 1000 m^30,41^. It should be noted that upwelling frequency and seasonality—and consequently their influence on timing of diapause in *C. natalis* C5s—differ between the nBUS, where upwelling is more persistent but diffuse throughout most of the year, and the sBUS, where it is more strongly pulsed and confined to the upwelling season.

The abundances of different life stages of *C. natalis* have been studied under contrasting upwelling conditions at an inshore anchor station in St Helena Bay (32°S)^57^, located close to the coastal station of our ST3 transect. In that study, abundances of C4 to adult stages were in a similar range as in the present study (35,735 vs. 38,635 ind m^−2^, resp.). Interestingly, the total abundance of *C. natalis* inshore along ST3 was only slightly higher than that reported from more than three decades earlier^57^. This was unexpected as the copepod community structure in the sBUS had shifted from a dominance of medium-sized to large species in the 1980s to a dominance of smaller species in the 2000s, and *C. natalis* belongs to the larger size category^13^. Following an overall increase of zooplankton in the sBUS since the 1950s, abundance decreased again since the mid-1990s^13^. However, with the recovery and reestablished dominance of sardines in the early 2010s^47^ the anchovy stocks declined again in the sBUS and were fairly low in 2019^61^, when our copepod samples were collected. Thus, predation pressure by anchovies on their preferred prey species such as *C. natalis*^41^ was probably lower during our sampling time compared to the study in 1984^57^, potentially explaining the higher abundances of *C. natalis* observed in the present study.

Compared to anchovy, sardines prefer smaller prey items^10^. Potential predation by sardines on early copepodite stages C1-3 of *C. natalis* was likely low, too. Since the mid-2000s, the sardine population has remained low and depleted in the sBUS^62^. SST and copepod abundance have been identified as the most influential environmental variables affecting sardine growth^31^. Sardines spawn in autumn or spring on the Agulhas Bank. Eggs and larvae are either retained on the Agulhas Bank or transported to the west coast nursery ground, where pre-recruits (20–50 mm length) initially remain offshore. As juveniles (60–80 mm length), they migrate towards the productive inshore region of the west coast between 29°S and 34°S, where they actively feed on phytoplankton and small copepods^31,47^.

In our study, females and males of *C. natalis* usually occurred in subsurface layers (50–100 m) during daytime and in surface layers (0–50 m) at night time stations. Previous studies reported small-scale diel vertical migration (DVM) behavior, i.e. within the upper 200 m^57^. At daytime stations in the sBUS with low chl *a* concentrations, *C. natalis* was most abundant in both surface and subsurface layers. Reduced phytoplankton availability may limit feeding success in surface waters and thereby diminish DVM^44^. High abundances of deep-dwelling *C. natalis* C5s in the nBUS reflect ontogenetic vertical migration (OVM) associated with end-of-season diapause^63^. The cessation of upwelling prevents the inshore advection of offshore populations^28,64^. The low abundance of deep *C. natalis* C5s at ST1 suggests that most of the offshore population had been transported inshore by the active upwelling front^28,64^, leaving few individuals offshore at depth.

**Fig. 11:**
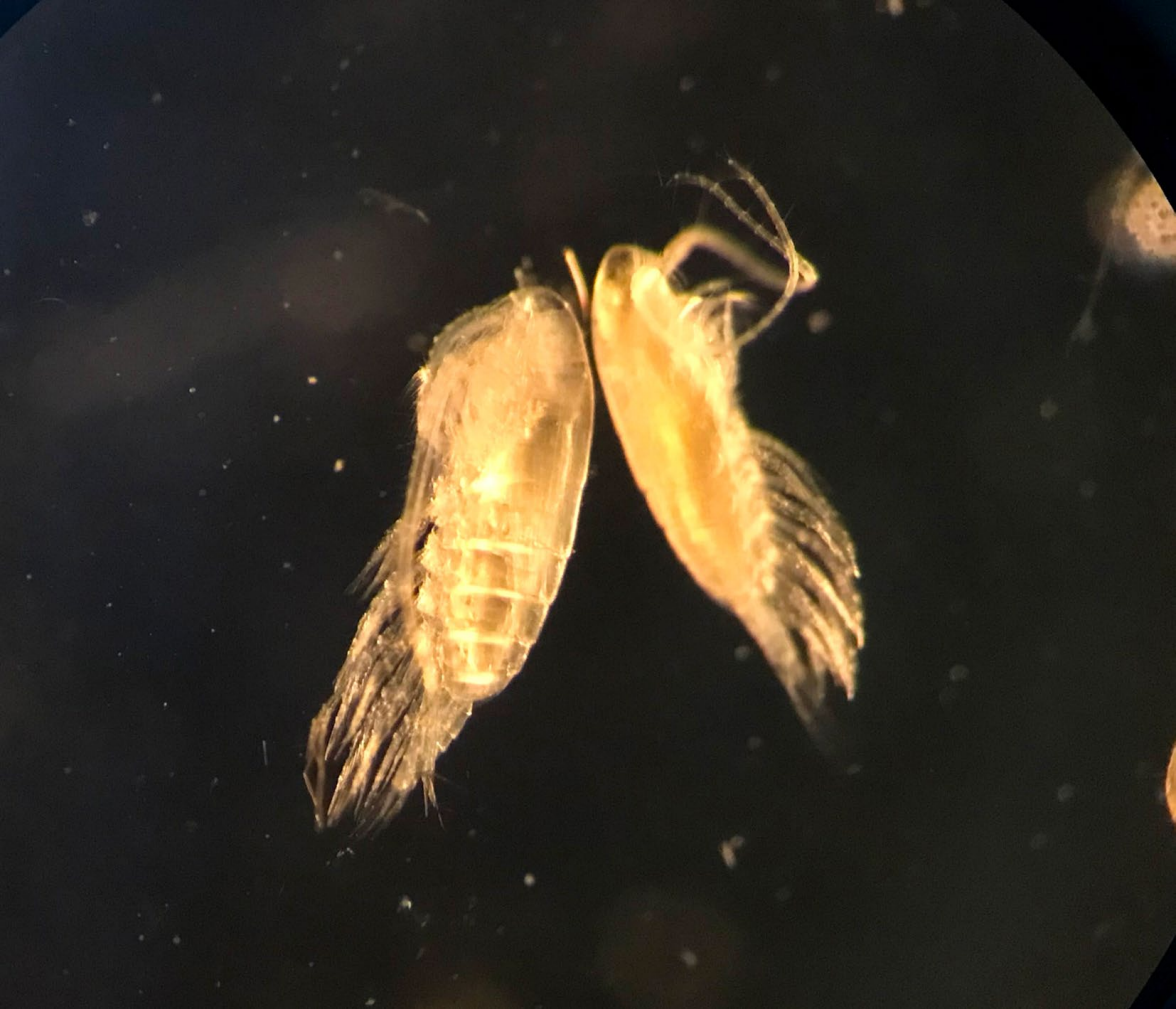
Habitus of adult females of *Calanus agulhensis* (left) and *Calanoides natalis* (right) showing large lipid sacs in their bodies.

Likewise, small-scale DVM of *C. agulhensis* was observed for copepodids C4, C5 and adult females along the South African west coast^44^ when chl *a* concentrations in coastal areas were usually high. OVM has so far not been decribed for *C. agulhensis*, since, to our knowledge, previous studies on this species focused on the epipelagic zone^23,44^. Similar to *C. natalis* in this study, deep-dwelling C5s of C*. agulhensis* were present along all transects, typically below 200 m in the sBUS and below 400 m in the nBUS, where they occurred only at very low abundance. High abundances of these C5s at depth in the sBUS, together with large, golden lipid sacs observed in deep-dwelling individuals under a dissecting microscope (Fig. 11) and elevated levels of WEs, indicate that the life cycle of *C. agulhensis* includes a form of diapause or dormant stage at depth. It has been suggested that *C. agulhensis* does not perform diapause during its life cycle^44^, however, observations were based on samples collected exclusively from the upper 200 m on the Agulhas Bank^44,63^. Our study is the first documented instance when copepodids C5 of *C. agulhensis* were collected at mesopelagic depths and their lipid compositions were analyzed. Their elevated WE levels raise questions regarding their potential life cycle adaptations, e.g., whether they are able to enter into diapause at depth and, if so, why they cannot establish larger population sizes on the shelf similar to *C. natalis*. It is apparent that they are outcompeted by *C. natalis* in both the nBUS and the sBUS, possibly connected to *C. natalis*’ higher preference for consuming diatoms, as reflected in their higher levels of diatom marker FAs^44,63^. *C. agulhensis* belongs to the *helgolandicus* lineage of *Calanus*, which comprises several species exhibiting functional traits resembling those of *C. natalis*^65^. Other members of this lineage, such as the North Atlantic *Calanus finmarchicus*, are known to enter diapause at copepodite stage C5 at depths between 500 and 1000 m^65,66^. Our sample size was too limited to draw generalized conclusions, but will encourage future studies to explore the life cycle of *C. agulhensis* in the BUS. Elucidating differences in functional traits and life cycle adaptations of key prey species will advance our understanding of how such differences translate up the food web into fish stocks.

## Conclusions

Within each size category of the six Calanidae species studied, closely related species exhibited similar functional traits, yet each species pair differed in at least one niche—either horizontal or vertical distribution and/or dietary spectra. *C. natalis* was associated with colder, chl *a*-rich shelf waters and showed strong diatom feeding, effectively outcompeting *C. agulhensis* in the study area. In contrast, *C. agulhensis* was linked to warmer offshore waters and was adapted to lower, more stable food concentrations.

Future intensification of upwelling in certain regions due to climate change-driven enforcement of wind fields may favor *C. natalis*, which is well adapted to coastal upwelling conditions, i.e. high but very variable food levels, preferably diatoms. An increase in *C. natalis* abundance could propagate up the food web, benefiting mid-trophic level clupeoid fishes such as anchovies that feed on larger copepods^31,62^. The threshold of copepod biomass required to sustain large anchovy stocks remains unknown, as their populations appeared depleted during our 2019 sampling^61^. However, the 2023 status report of South African marine fishery resources states that anchovy stocks have returned to optimal levels in the sBUS, whereas sardine stocks remain below the long-term average^67^, suggesting that *C. natalis* biomass has recently been sufficient to support reasonable anchovy populations. The present study also highlights the crucial role of diatoms at the base of highly productive marine ecosystems, both for energy transfer and the biological carbon pump, underscoring the need for interdisciplinary monitoring studies linking lower to higher trophic levels. It is still uncertain that coastal upwelling in the BUS and other Eastern Boundary Upwelling Systems will intensify due to anthropogenic climate change^68,69^.

Upwelling systems are characterized by a “wasp-waist” food web with a limited number of mid-trophic species, such as the three small pelagic clupeoids in the sBUS, *Engraulis capensis*, *Etrumeus whiteheadi*, and *Sardinops sagax*^70^, which serve as key conduits of energy from lower to higher trophic levels^71^. The interconnectedness of copepod biomass, distribution and community structure to fish stock dynamics is not yet fully understood. For example, it remains unclear whether high abundances of *C. agulhensis* on the Agulhas Bank are associated with high-biomass anchovy years, highlighting the prediction potential of zooplankton monitoring^23,31^. Addressing such questions requires closely coupled, long-term interdisciplinary research.

This study highlights that closely related, similar-sized copepod species possess highly specialized life-cycle adaptations and functional traits, making detailed investigations of zooplankton dynamics essential for understanding and ultimately predicting population dynamics of higher trophic levels, including commercially important fish.

## Material and methods

### Zooplankton sampling and environmental variables

Mesozooplankton samples were collected using a vertical multiple opening/closing net (HydroBios Multinet Midi: 0.25 m^2^ mouth opening, five nets, 200 µm mesh size, 0.5 m s^−1^ hauling speed) during expedition M153 aboard RV *Meteor* in the nBUS and sBUS from February 18 to March 8 2019 (Fig. 1). Two cross-shelf transects located in the nBUS (NT1, NT2) and three located in the sBUS (ST1, ST2, ST3) were analyzed. Vertically stratified hauls were taken from 10 m above the sea floor on the shelf and from a maximum depth of 1500 m further offshore (see 10.1594/PANGAEA.983970). At inshore stations, shallow bottom depths < 80 m resulted in only two sampled depth intervals, while five depth intervals were sampled above the continental slope and further offshore. A calibrated flowmeter was attached into the mouth opening of the net frame to measure the filtered seawater volume. Most samples were preserved in a 4% borax-buffered formaldehyde seawater solution; a few samples were preserved in absolute ethanol. Samples were transported to Germany for further analysis.

Water column temperature, salinity, dissolved oxygen concentration and fluorescence were measured at each station with a conductivity temperature depth (CTD) profiling system (SEABIRD ELECTRONICS (SBE) 911PLUS), equipped with a DIGIQUARZ pressure sensor, a SBE3 temperature and SBE4 conductivity sensor with a double sensor setup, and a SBE43 dissolved oxygen sensor. Fluorescence provided a proxy for chl *a* concentration in the upper 200 m.

To identify differences in oceanographic parameters between the nBUS and the sBUS, a PCA was conducted based on environmental variables (quantitative supplements), i.e. mean temperature, salinity, fluorescence and oxygen concentration per sampled depth layer in R^72^ (version 4.2.2) using the FactoMineR package^73^. Environmental parameters were standardized and categorical variables (qualitative supplements) were added prior to PCA, i.e. subsystem (nBUS, sBUS), depth layer (upper/lower epipelagic, upper/lower mesopelagic, bathypelagic) and presence and absence of respective calanid species.

### Abundance analysis

Mesozooplankton samples were split in the home laboratory using a Motoda plankton splitter^74^. One half was used for abundance analysis under a dissecting microscope (Leica MZ12.5), the other half was stored for further studies. In terms of formalin-preserved samples, one half of the sample was transferred to a sorting fluid consisting of 0.5% propylene phenoxetol, 5% propylene glycol, and 94.5% freshwater^75^. Copepods of the family Calanidae were identified to species and developmental stages and enumerated until at least 80–100 individuals per category were counted within a subsample, usually requiring sample splitting to ¼ and up to 1/64 for abundant species. Specimens of rare species or stages were counted from the entire half of the sample. Calanid copepods were identified to species level according to identification keys^49,50^. Copepodite stages of calanid species were separated into copepodids C1-3, C4, C5, adult females and adult males (NT1, ST1, ST3). At NT2 and ST2, copepodids C4-5 were treated as a single category because they had already been analyzed as part of a separate study; the corresponding data (ST2 from^76^ and unpublished NT2 data) were incorporated into the present analysis. Copepodids C1-3 of Calanidae were highly abundant but difficult to assign quantitatively to species level and therefore pooled at family level into one category.

To determine differences in vertical distribution of species and copepodite stages, WMDs were calculated after^77^:

$$WMD = \frac{{\sum \left( {n_{i} *z_{i} *d_{i} } \right)}}{{\sum \left( {n_{i} *z_{i} } \right)}}$$

where n_i_ is abundance per m^3^, z_i_ is the depth range of the respective sampling interval and d_i_ is the mid-depth of the sampling interval.

To reveal distribution patterns the *metaMDS* function in the *vegan* package^78^ in R^72^ (version 4.4.2) was applied with (1) species-specific total abundances per station (ind m^−2^) and (2) with species- and stage-specific abundances per sampled depth layer (ind m^−3^). The *envfit* function was used to fit environmental variables on the ordination for (1) surface temperature (as proxy for upwelling intensity), salinity, fluorescence (as proxy for chl *a*), oxygen concentration of the top layer and bottom depth of the respective station and for (2) mean temperature, salinity, fluorescence, oxygen concentration, and sampling depth of each respective layer. Pearson correlation coefficients with significance levels were calculated using the *Hmisc* package^79^ and plotted as heatmap using the *reshape2*^80^ and *ggplot2* packages ^81^ in *R*.

### Stable isotope analysis

To compare δ^15^N ratios at high species and stage resolution, specimens were sorted from the formalin-preserved samples^82^. To avoid bias of baseline differences among stations, specimens were sorted from one station (stn. 18). Depending on their sizes, several specimens were pooled into one measurement to receive ~ 0.5–1.0 mg dry mass after lyophilization for 48 h per sample (Sartorius, NCII1S, precision ± 10 µg). Samples were transferred into tin capsules and stable isotope analysis was performed by TÜV Rheinland Agroisolab GmbH in Jülich, Germany, using a mass spectrometer (EA NA1500 Series 2, Carlo Erba Instruments) and helium as carrier gas. Stable isotope ratios of nitrogen (^15^N/^14^N) were measured against the IAEA-N1 standards and are expressed as δ^15^N in ‰ with respect to atmospheric air ^83^.

To test for differences in δ^15^N of formalin-preserved samples in comparison to frozen samples, also deep-frozen copepodid C5s of *C. natalis* were analyzed for δ^15^N that had been frozen (− 80 °C) at the same station (stn. 18). δ^15^N values of deep-frozen specimens from stn. 18 were lower for copepodids C5 of *C. natalis* with 9.1 ± 0.3‰ compared to 9.4 ± 0.6‰ of formalin-fixed samples and higher for frozen copepodids C5 of *C. agulhensis* with 10.0 ± 0.8‰ compared to 9.0 ± 0.2‰ of formalin-fixed samples.

### Fatty acid analysis

Immediately after each haul and prior to fixation, larger calanids were gently identified to species and stages (adult females, males, copepodite stage C5), enumerated and quickly deep-frozen at − 80 °C for biochemical analysis in the home laboratory. A second cruise with RV *Sonne* (SO285) with the same sampling scheme took place in September 2021, from which deep-frozen samples were added to the FA analyses. Since FA compositions are more robust to local influences^84^, samples for lipid analysis were taken from different stations to increase sample size and number of replicates (10.1594/PANGAEA.983970). Body dry mass (DM) of the deep-frozen calanids was determined after lyophilization for 48 h. Several individuals were pooled to obtain sufficient biomass (> 1 mg DM). Lipids were extracted with dichloromethane:methanol (2:1, v:v) and purified by adding a 0.88% aqueous KCl solution^85^. The total lipid (TL) content was determined gravimetrically and is given in percent of DM.

Lipids were converted to their FA methyl ester (FAME) derivatives by transesterification in methanol with 3% concentrated sulfuric acid ^86^. Subsequently, FAs and fatty alcohols (FAlc) were quantified using a gas chromatograph (Agilent Technologies 7890A) equipped with a DB-FFAP column (30 m length, 0.25 mm diameter) using a programmable temperature vaporizer injector and helium as carrier gas. FAs and FAlcs were routinely identified by comparing their retention times with those of natural standards (Supelco® 37 Component FAME Mix and *Calanus hyperboreus*) of known composition. The portion of WEs was calculated based on FAlc content assuming equal masses of the FA and FAlc moieties in the WE molecules and is given as percent of TL^87^.

FA trophic biomarkers such as 16:1(n-7), 16:4(n-1) and 18:1(n-7) indicate a diatom-dominated food source, while 18:4(n-3) is commonly used as a dinoflagellate biomarker^88^. In contrast, 18:1(n-9) is known as a carnivory biomarker. The carnivory index (CI), a proportional measure of the carnivorous contribution to the copepods’ diets^84^, was calculated as CI = 18:1(n-9)/((16:1(n-7) + 16:4(n-1) + 18:1(n-7) + 18:4(n-3) + 18:1(n-9)). In addition, the bacterial contribution (BI) to the nutrition of consumers was calculated as the sum of odd-numbered (15:0, 17:0) and *iso*-branched fatty acids (*iso* 15:0, *iso* 17:0), i.e. BI = Σbacterial markers/(Σherbivorous markers + bacterial markers)^84^.

To identify differences in the dietary composition of calanid species and copepodite stages, a PCA was conducted based on FA composition data including FAs > 1% of total FAs using the *Primer v6* software^89^. Prior to PCA, proportions of FAs were arcsine-square-root transformed to correct deficiencies in normality and homogeneity of variance.

## Data Availability

Data sets are available in PANGAEA: 10.1594/PANGAEA.983970.

## References

[1] Hutchings, L. et al. The Benguela Current: An ecosystem of four components. *Prog. Oceanogr.* **83**, 15–32 (2009).

[2] Crawford, R. J. M., Shannon, L. V. & Pollock, D. E. The Benguela ecosystem part VI. The major fish and invertebrate resources. *Oceanogr. Mar. Biol. Annu. Rev.* **25**, 323–505 (1987).

[3] Crawford, R. J. M. Food, fishing and seabirds in the Benguela upwelling system. *J. Ornithol.* **148**, S253–S260 (2007).

[4] Ekau, W. et al. Pelagic key species and mechanisms driving energy flows in the northern Benguela upwelling ecosystem and their feedback into biogeochemical cycles. *J. Mar. Syst.* **188**, 49–62 (2018).

[5] Geist, S. J. et al. Distribution, feeding behaviour, and condition of Cape horse mackerel early life stages, *Trachurus capensis*, under different environmental conditions in the northern Benguela upwelling ecosystem. *ICES J. Mar. Sci.* **72**, 543–557 (2015).

[6] Kirkman, S. P. et al. Spatial characterisation of the Benguela ecosystem for ecosystem-based management. *Afr. J. Mar. Sci.* **38**, 7–22 (2016).

[7] Roux, J.-P. et al. Jellyfication of marine ecosystems as a likely consequence of overfishing small pelagic fishes: Lessons from the Benguela. *Bull. Mar. Sci.* **89**, 249–284 (2013).

[8] Watermeyer, K., Shannon, L., Roux, J.-P. & Griffiths, C. Changes in the trophic structure of the northern Benguela before and after the onset of industrial fishing. *Afr. J. Mar. Sci.* **30**, 383–403 (2008).

[9] Coetzee, J. C., van der Lingen, C. D., Hutchings, L. & Fairweather, T. P. Has the fishery contributed to a major shift in the distribution of South African sardine?. *ICES J. Mar. Sci.* **65**, 1676–1688 (2008).

[10] van der Lingen, C. D., Hutchings, L. & Field, J. G. Comparative trophodynamics of anchovy *Engraulis encrasicolus* and sardine *Sardinops sagax* in the southern Benguela: Are species alternations between small pelagic fish trophodynamically mediated?. *Afr. J. Mar. Sci.* **28**, 465–477 (2006).

[11] van der Lingen, C. D. & Hampton, I. Climate change impacts, vulnerabilities and adaptations: Southeast Atlantic and Southwest Indian Ocean marine fisheries. In *Impacts of Climate Change on Fisheries and Aquaculture* (eds. Brange, M. et al.) 219–250 (FAO Fisheries and Aquaculture Technical Paper, Rome, 2018).

[12] Shannon, L. J., Moloney, C. L., Jarre, A. & Field, J. G. Trophic flows in the southern Benguela during the 1980s and 1990s. *J. Mar. Syst.* **39**, 83–116 (2003).

[13] Verheye, H. M., Lamont, T., Huggett, J. A., Kreiner, A. & Hampton, I. Plankton productivity of the Benguela Current Large Marine Ecosystem (BCLME). *Environ. Dev.* **17**, 75–92 (2016).

[14] Verheye, H. M., Hutchings, L., Huggett, J. A. & Painting, S. J. Mesozooplankton dynamics in the Benguela ecosystem, with emphasis on the herbivorous copepods. *S. Afr. J. Mar. Sci.* **12**, 561–584 (1992).

[15] Hansen, F. C., Cloete, R. R. & Verheye, H. M. Seasonal and spatial variability of dominant copepods along a transect off Walvis Bay (23°S), Namibia. *Afr. J. Mar. Sci.* **27**, 55–63 (2005).

[16] Bode, M. et al. Spatio-temporal variability of copepod abundance along the 20°S monitoring transect in the northern Benguela Upwelling System from 2005 to 2011. *PLoS ONE* **9**, e97738 (2014). 24844305 10.1371/journal.pone.0097738 PMC4028300

[17] Emeis, K. et al. Biogeochemical processes and turnover rates in the Northern Benguela Upwelling System. *J. Mar. Syst.* **188**, 63–80 (2018).

[18] Mohrholz, V. et al. Cross shelf hydrographic and hydrochemical conditions and their short-term variability at the northern Benguela during a normal upwelling season. *J. Mar. Syst.* **140**, 92–110 (2014).

[19] Bertrand, A. et al. Oxygen: A fundamental property regulating pelagic ecosystem structure in the coastal southeastern tropical Pacific. *PLoS ONE* **6**, e29558 (2011). 22216315 10.1371/journal.pone.0029558 PMC3247266

[20] Ekau, W., Auel, H., Pörtner, H.-O. & Gilbert, D. Impacts of hypoxia on the structure and processes in pelagic communities (zooplankton, macro-invertebrates and fish). *Biogeosciences* **7**, 1669–1699 (2010).

[21] Auel, H. & Verheye, H. M. Hypoxia tolerance in the copepod *Calanoides carinatus* and the effect of an intermediate oxygen minimum layer on copepod vertical distribution in the northern Benguela Current upwelling system and the Angola–Benguela Front. *J. Exp. Mar. Biol. Ecol.* **352**, 234–243 (2007).

[22] Wishner, K. F., Seibel, B. & Outram, D. Ocean deoxygenation and copepods: Coping with oxygen minimum zone variability. *Biogeosciences* **17**, 2315–2339 (2020).

[23] Huggett, J. A., Noyon, M., Carstensen, J. & Walker, D. R. Patterns in the plankton—Spatial distribution and long-term variability of copepods on the Agulhas Bank. *Deep Sea Res. Part II Top. Stud. Oceanogr.*10.1016/j.dsr2.2023.105265 (2023).

[24] Huggett, J. A., Richardson, A. J. & Field, J. G. Comparative ecology of the copepods *Calanoides carinatus* and *Calanus agulhensis*—The influence of temperature and food. *Afr. J. Mar. Sci.* **29**, 473–490 (2007).

[25] Auel, H., Hagen, W., Ekau, W. & Verheye, H. M. Metabolic adaptations and reduced respiration of the copepod *Calanoides carinatus* during diapause at depth in the Angola-Benguela Front and northern Benguela upwelling regions. *Afr. J. Mar. Sci.* **27**, 653–657 (2005).

[26] Verheye, H. M. et al. Life strategies, energetics and growth characteristics of *Calanoides carinatus* (Copepoda) in the Angola-Benguela frontal region. *Afr. J. Mar. Sci.* **27**, 641–651 (2005).

[27] Kosobokova, K. N., Drits, A. V. & Krylov, P. Physiological and biochemical characteristics of *Calanoides carinatus* in waters of an upwelling off coast of Namibia. *Oceanology* **28**, 375–379 (1988).

[28] Verheye, H. M. Short-term variability during an anchor station study in the southern Benguela upwelling system: Abundance, distribution and estimated production of mesozooplankton with special reference to *Calanoides carinatus* (Krøyer, 1849). *Prog. Oceanogr.* **28**, 91–119 (1991).

[29] Arashkevich, E. G., Drits, A. V. & Timonin, A. G. Diapause in the life cycle of *Calanoides carinatus* (Krøyer), (Copepoda, Calanoida). *Hydrobiologia* **320**, 197–208 (1996).

[30] Loick, N., Ekau, W. & Verheye, H. Water-body preferences of dominant calanoid copepod species in the Angola-Benguela frontal zone. *Afr. J. Mar. Sci.* **27**, 597–608 (2005).

[31] Brinkman, F. R. V. et al. Unveiling ecosystem shifts in the southern Benguela through otolith biochronologies of sardine (*Sardinops sagax*). *Fish. Oceanogr.* **34**, e12710 (2024).

[32] Shannon, L. V. et al. The 1980s—A decade of change in the Benguela ecosystem. *South Afr. J. Mar. Sci.* **12**, 271–276 (1992).

[33] Verheye, H. M. Decadal-scale trends across several marine trophic levels in the southern Benguela Upwelling System off South Africa. *AMBIO J. Hum. Environ.* **29**, 30–34 (2000).

[34] Verheye, H. M. & Richardson, A. J. Long-term increase in crustacean zooplankton abundance in the southern Benguela upwelling region (1951–1996): Bottom-up or top-down control?. *ICES J. Mar. Sci.* **55**, 803–807 (1998).

[35] Lamont, T., Garcia-Reyes, M., Bograd, S. J., van der Lingen, C. D. & Sydeman, W. J. Upwelling indices for comparative ecosystem studies: Variability in the Benguela Upwelling System. *J. Mar. Syst.* **188**, 3–16 (2018).

[36] Rouault, M., Pohl, B. & Penven, P. Coastal oceanic climate change and variability from 1982 to 2009 around South Africa. *Afr. J. Mar. Sci.* **32**, 237–246 (2010).

[37] Verheye, H. M., Richardson, A. J., Hutchings, L., Marska, G. & Gianakouras, D. Long-term trends in the abundance and community structure of coastal zooplankton in the southern Benguela system, 1951–1996. *South Afr. J. Mar. Sci.* **19**, 317–332 (1998).

[38] Rixen, T. et al. Oxygen and nutrient trapping in the Southern Benguela Upwelling System. *Front. Mar. Sci.* **8**, 730591 (2021).

[39] Gibbons, M. J. & Hutchings, L. Zooplankton diversity and community structure around southern Africa, with special attention to the Benguela upwelling system. *South Afr. J. Sci.* **92**, 63–75 (1996).

[40] Schukat, A., Teuber, L., Hagen, W., Wasmund, N. & Auel, H. Energetics and carbon budgets of dominant calanoid copepods in the northern Benguela upwelling system. *J. Exp. Mar. Biol. Ecol.* **442**, 1–9 (2013).

[41] Verheye, H. M., Hutchings, L. & Peterson, W. T. Life history and population maintenance strategies of *Calanoides carinatus* (Copepods: Calanoida) in the southern Benguela ecosystem. *South Afr. J. Mar. Sci.* **11**, 179–191 (1991).

[42] Benedetti, F., Gasparini, S. & Ayata, S.-D. Identifying copepod functional groups from species functional traits. *J. Plankton Res.* **38**, 159–166 (2016). 26811565 10.1093/plankt/fbv096 PMC4722884

[43] Brun, P., Payne, M. R. & Kiørboe, T. A trait database for marine copepods. *Earth Syst. Sci. Data* **9**, 99–113 (2017).

[44] Huggett, J. A. & Richardson, A. J. A review of the biology and ecology of *Calanus agulhensis* off South Africa. *ICES J. Mar. Sci.* **57**, 1834–1849 (2000).

[45] Morgan, C. A., Peterson, W. T. & Emmett, R. L. Onshore-offshore variations in copepod community structure off the Oregon coast during the summer upwelling season. *Mar. Ecol. Prog. Ser.* **249**, 223–236 (2003).

[46] Schukat, A., Auel, H., Teuber, L. & Hagen, W. Complex trophic interactions of calanoid copepods in the Benguela upwelling system. *J. Sea Res.* **85**, 186–196 (2014).

[47] Hutchings, L., Jarre, A., Lamont, T., van den Berg, M. & Kirkman, S. P. St Helena Bay (southern Benguela) then and now: Muted climate signals, large human impact. *Afr. J. Mar. Sci.* **34**, 559–583 (2012).

[48] Bradford-Grieve, J. M. & Ahyong, S. T. Phylogenetic relationships among genera in the Calanidae (Crustacea: Copepoda) based on morphology. *J. Nat. Hist.* **44**, 279–299 (2010).

[49] Bradford-Grieve, J. M., Markhaseva, E., Rocha, C. E. F. & Abiahy, B. B. Copepoda. In *South Atlantic Zooplankton* Vol. 2 (ed. Boltovskoy, D.) 869–1098 (Backhuys Publishers, Leiden, 1999).

[50] Razouls, C., Desreumanaux, N., Kouwenberg, J., & de Bovée, F. Biodiversity of marine planktonic copepods (morphology, geographical distribution and biological data). http://copepodes.obs-banyuls.fr/en (2005).

[51] Shimode, S., Hiroe, Y., Hidaka, K., Takahashi, K. & Tsuda, A. Life history and ontogenetic vertical migration of *Neocalanus gracilis* in the western North Pacific Ocean. *Aquat. Biol.* **7**, 295–306 (2009).

[52] Atkinson, A. & Sinclair, J. D. Zonal distribution and seasonal vertical migration of copepod assemblages in the Scotia Sea. *Polar Biol.* **23**, 46–58 (2000).

[53] Miller, K. J., Bradford-Grieve, J. M. & Jillett, J. B. Genetic relationship between winter deep-dwelling and spring surface-dwelling female *Neocalanus tonsus* in the Southern Ocean. *Mar. Biol.* **134**, 99–106 (1999).

[54] Ohman, M. D., Bradford, J. M. & Jillett, J. B. Seasonal growth and lipid storage of the circumglobal, subantarctic copepod, *Neocalanus tonsus* (Brady). *Deep Sea Res. Part A Oceanogr. Res. Pap.* **36**, 1309–1326 (1989).

[55] Richardson, A. J., Verheye, H. M., Herbert, V., Rogers, C. & Arendse, L. M. Egg production, somatic growth and productivity of copepods in the Benguela Current system and Angola-Benguela Front. *South Afr. J. Mar. Sci.* **97**, 251–257 (2001).

[56] Ramírez, F. C. & Sabatini, M. E. The occurrence of Calanidae species in waters off Argentina. *Hydrobiologia* **439**, 21–42 (2000).

[57] Verheye, H. M. & Field, J. G. Vertical distribution and diel vertical migration of *Calanoides carinatus* (Krøyer, 1849) developmental stages in the southern Benguela upwelling region. *J. Exp. Mar. Biol. Ecol.* **158**, 123–140 (1992).

[58] Hirst, A. G., Bonnet, D., Conway, D. V. P. & Kiørboe, T. Does predation control adult sex ratios and longevities in marine pelagic copepods?. *Limnol. Oceanogr.* **55**, 2193–2206 (2010).

[59] Titelman, J., Varpe, O., Eliassen, S. & Fiksen, O. Copepod mating: Chance or choice?. *J. Plankton Res.* **29**, 1023–1030 (2007).

[60] Kiørboe, T. Sex, sex-ratios, and the dynamics of pelagic copepod populations. *Oecologia* **148**, 40–50 (2006). 16425044 10.1007/s00442-005-0346-3

[61] DEFF (Department of Environment, Forestry and Fisheries) 2020. *Status of the South African Marine Fishery Resources 2020*. 10.13140/RG.2.2.30734.48962.

[62] van der Lingen, C. D. Adapting to climate change in the South African small pelagic fishery. In *Adaptive Management of Fisheries in Response to Climate Change* (eds. Bahri, T. et al.) 177–194 (Food and Agriculture Organization of the United Nations (FAO), Rome, 2021).

[63] Peterson, W. Life cycle strategies of copepods in coastal upwelling zones. *J. Mar. Syst.* **15**, 313–326 (1998).

[64] Höring, F., Cornils, A., Auel, H., Bode, M. & Held, C. Population genetic structure of *Calanoides natalis* (Copepoda, Calanoida) in the eastern Atlantic Ocean and Benguela upwelling system. *J. Plankton Res.* **39**, 618–630 (2017).

[65] Sabatini, M., Ramírez, F. C. & Martos, P. Distribution pattern and population structure of *Calanus australis* Brodsky, 1959 over the southern Patagonian Shelf off Argentina in summer. *ICES J. Mar. Sci.* **57**, 1856–1866 (2000).

[66] Saumweber, W. J. & Durbin, E. G. Estimating potential diapause duration in *Calanus finmarchicus*. *Deep Sea Res. Part II Top. Stud. Oceanogr.* **53**, 2597–2617 (2006).

[67] DFFE (Department of Forestry, Fisheries and the Environment). *Status of the South African Marine Fishery Resources 2023*. 10.15493/DFFE.10000006 (2023).

[68] Tim, N., Zorita, E. & Hünicke, B. Decadal variability and trends of the Benguela upwelling system as simulated in a high-resolution ocean simulation. *Ocean Sci.* **11**, 483–502 (2015).

[69] Bograd, S. J. et al. Climate change impacts on eastern boundary upwelling systems. *Annu. Rev. Mar. Sci.* **15**, 303–328 (2023). 10.1146/annurev-marine-032122-021945 35850490

[70] Cury, P. Small pelagics in upwelling systems: Patterns of interaction and structural changes in “wasp-waist” ecosystems. *ICES J. Mar. Sci.* **57**, 603–618 (2000).

[71] van der Lingen, C. D. Diet of sardine *Sardinops sagax* in the southern Benguela upwelling ecosystem. *S. Afr. J. Mar. Sci.* **24**, 301–316 (2002).

[72] R Core Team. R: A Language and Environment for Statistical Computing. R Foundation for Statistical Computing. (2019).

[73] Lê, S., Josse, J. & Husson, F. FactoMineR: An R package for multivariate analysis. *J. Stat. Softw.* **25**, 1–18 (2008).

[74] Motoda, S. Instructions for use devices of simple plankton apparatus Faculty of Fisheries, Hokkaido University. *Mem. Fac. Fish. Hokkaido Univ.* **7**, 73–111 (1959).

[75] Steedman, H. F. Zooplankton fixation and preservation. In *Monographs Oceangraphic Methology*, Vol. 4 (UNESCO Press, Vendome, 1976).

[76] Bode-Dalby, M. et al. Small is beautiful: The important role of small copepods in carbon budgets of the southern Benguela upwelling system. *J. Plankton Res.* **45**, 110–128 (2023).

[77] Andersen, V., Gubanova, A., Nival, P. & Ruellet, T. Zooplankton community during the transition from spring bloom to oligotrophy in the open NW Mediterranean and effects of wind events. 2. Vertical distributions and migrations. *J. Plankton Res.* **23**, 243–261 (2001).

[78] Oksanen, J. et al. vegan: Community Ecology Package. 2.6-10 10.32614/CRAN.package.vegan (2019).

[79] Harrell, J., F. E. Hmisc: Harrell Miscellaneous. 5.2–3 10.32614/CRAN.package.Hmisc (2025).

[80] Wickham, H. reshape2: Flexibly reshape data: A reboot of the reshape package. 1.4.4 10.32614/CRAN.package.reshape2 (2010).

[81] Wickham, H. *Ggplot2: Elegant Graphics for Data Analysis* (Springer, 2016).

[82] Bicknell, A. W. J. et al. Effects of formalin preservation on stable carbon and nitrogen isotope signatures in calanoid copepods: Implications for the use of Continuous Plankton Recorder Survey samples in stable isotope analyses. *Rapid Commun. Mass Spectrom.* **25**, 1794–1800 (2011). 21638354 10.1002/rcm.5049

[83] Hobson, K. A. et al. A stable isotope (δ^13^C, δ^15^N) model for the North Water food web: Implications for evaluating trophodynamics and the flow of energy and contaminants. *Deep Sea Res. Part II Top. Stud. Oceanogr.* **49**, 5131–5150 (2002).

[84] Bode, M. et al. Feeding strategies of tropical and subtropical calanoid copepods throughout the eastern Atlantic Ocean—Latitudinal and bathymetric aspects. *Prog. Oceanogr.* **138**, 268–282 (2015).

[85] Folch, J., Lees, M. & Sloane Stanley, G. H. A simple method for the isolation and purification of total lipides from animal tissues. *J. Biol. Chem.* **226**, 497–509 (1957). 13428781

[86] Kattner, G. & Fricke, H. S. G. Simple gas-liquid chromatographic method for the simultaneous determination of fatty acids and alcohols in wax esters of marine organisms. *J. Chromatogr. A* **361**, 263–268 (1986).

[87] Kattner, G., Albers, C., Graeve, M. & Schnack-Schiel, S. B. Fatty acid and alcohol composition of the small polar copepods, *Oithona* and *Oncaea*: Indication on feeding modes. *Polar Biol.* **26**, 666–671 (2003).

[88] Dalsgaard, J., St. John, M., Kattner, G., Müller-Navarra, D. & Hagen, W. Fatty acid trophic markers in the pelagic marine environment. *Adv. Mar. Biol.* **46**, 225–340 (2003). 14601414 10.1016/s0065-2881(03)46005-7

[89] Clarke, K. R. & Warwick, R. M. *Change in Marine Communities: An Approach to Statistical Analysis and Interpretation* 2nd edn. (PRIMER-E, Plymouth, 2001).

